# Posicionamento sobre o Manejo de Antitrombóticos e Anticoagulantes em Dengue – 2026

**DOI:** 10.36660/abc.20260216

**Published:** 2026-04-28

**Authors:** Fabio Grunspun Pitta, Matheus de Oliveira Laterza Ribeiro, Antonio Eduardo Pesaro, Camila Talita Machado Barbosa, Cláudio Humberto Gonçalves Maia, Daniella Motta da Costa Dan, Evelin Meline Lubrigati, Felipe Carvalho Oliveira, João Gabriel Batista Lage, Julia Nobrega de Brito, Leticia Neves Solon Carvalho, Luciana Dornfeld Bichuette, Luka David Lechinewski, Marcelle Gonçalves Henriques Lizandro, Marcelo Bettega, Marco Antonio Freitas de Queiroz Mauricio, Maria Antonieta Albanez A de Medeiros Lopes, Pedro Felipe Gomes Nicz, Pedro Henrique de Moraes Cellia, Sarah Fagundes Grobe, Sayuri Inuzuka, Stefano Garzon, Tannas Jatene, Wilton Francisco Gomes, Carlos Vicente Serrano

**Affiliations:** 1 Einstein Hospital Israelita São Paulo SP Brasil Einstein Hospital Israelita, São Paulo, SP – Brasil; 2 Instituto do Coração InCor SP Brasil Instituto do Coração InCor, SP – Brasil; 3 Instituto de Cardiologia e Transplantes do Distrito Federal Brasília DF Brasil Instituto de Cardiologia e Transplantes do Distrito Federal, Brasília, DF – Brasil; 4 Hospital Meridional de Cariacica Cariacica ES Brasil Hospital Meridional de Cariacica, Cariacica, ES – Brasil; 5 Hospital INC Curitiba PR Brasil Hospital INC, Curitiba, PR – Brasil; 6 Hospital Aliança Star – Rede D’Or Salvador BA Brasil Hospital Aliança Star – Rede D’Or, Salvador, BA – Brasil; 7 Hospital Universitário Clementino Fraga Filho da Universidade Federal do Rio de Janeiro Rio de Janeiro RJ Brasil Hospital Universitário Clementino Fraga Filho da Universidade Federal do Rio de Janeiro, Rio de Janeiro, RJ – Brasil; 8 Hospital dos Servidores do Estado de Pernambuco Recife PE Brasil Hospital dos Servidores do Estado de Pernambuco, Recife, PE – Brasil; 9 Real Hospital Português de Beneficência em Pernambuco Recife PE Brasil Real Hospital Português de Beneficência em Pernambuco, Recife, PE – Brasil; 10 Hospital de Messejana Dr Carlos Alberto Studart Gomes Fortaleza CE Brasil Hospital de Messejana Dr. Carlos Alberto Studart Gomes, Fortaleza, CE – Brasil; 11 Pontifícia Universidade Católica do Paraná Curitiba PR Brasil Pontifícia Universidade Católica do Paraná, Curitiba, PR – Brasil; 12 Hospital São Marcelino Champagnat Curitiba PR Brasil Hospital São Marcelino Champagnat, Curitiba, PR – Brasil; 13 Hospital DF Star Rede D’Or São Luiz Brasília DF Brasil Hospital DF Star - Rede D’Or São Luiz, Brasília, DF – Brasil; 14 Universidade Federal do Paraná Curitiba PR Brasil Universidade Federal do Paraná, Curitiba, PR – Brasil; 15 Universidade Federal do Espírito Santo Vitória ES Brasil Universidade Federal do Espírito Santo, Vitória, ES – Brasil; 16 Hospital Marcelino Champagnat Curitiba PR Brasil Hospital Marcelino Champagnat, Curitiba, PR – Brasil; 17 Hospital Universitário Cajuru Curitiba PR Brasil Hospital Universitário Cajuru, Curitiba, PR – Brasil; 18 Universidade Federal de Goiás Goiânia GO Brasil Universidade Federal de Goiás, Goiânia, GO – Brasil; 19 Einstein Hospital Israelita Goiânia GO Brasil Einstein Hospital Israelita, Goiânia, GO – Brasil; 20 Faculdades Pequeno Príncipe Curitiba PR Brasil Faculdades Pequeno Príncipe, Curitiba, PR – Brasil

**Table:** 

Posicionamento sobre o Manejo de Antitrombóticos e Anticoagulantes em Dengue – 2026	
O relatório abaixo lista as declarações de interesse conforme relatadas à SBC pelos especialistas durante o período de desenvolvimento deste posicionamento, 2025/2026.	
Especialista	Tipo de relacionamento com a indústria
Antônio Eduardo Pesaro	Nada a ser declarado
Camila Talita Machado Barbosa	Nada a ser declarado
Carlos Vicente Serrano Jr.	Nada a ser declarado
Cláudio Humberto Gonçalves Maia	Declaração financeira Outros relacionamentos Vínculo empregatício com a indústria farmacêutica, de órteses, próteses, equipamentos e implantes, brasileiras ou estrangeiras, assim como se tem relação vínculo empregatício com operadoras de planos de saúde ou em auditorias médicas (incluindo meio período) durante o ano para o qual você está declarando: - Auditor médico de uma operadora de auto-gestão em saúde (plano de saúde dos servidores da Câmara Legislativa do Distrito Federal) - Serviço público.
Daniella Motta da Costa Dan	Declaração financeira Outros relacionamentos Financiamento de atividades de educação médica continuada, incluindo viagens, hospedagens e inscrições para congressos e cursos, provenientes da indústria farmacêutica, de órteses, próteses, equipamentos e implantes, brasileiras ou estrangeiras: - Viatris: Inspra.
Evelin Meline Lubrigati	Nada a ser declarado
Fabio Grunspun Pitta	Nada a ser declarado
Felipe Carvalho de Oliveira	Nada a ser declarado
João Gabriel Batista Lage	Nada a ser declarado
Julia Nobrega de Brito	Declaração financeira A - Pagamento de qualquer espécie e desde que economicamente apreciáveis, feitos a (i) você, (ii) ao seu cônjuge/ companheiro ou a qualquer outro membro que resida com você, (iii) a qualquer pessoa jurídica em que qualquer destes seja controlador, sócio, acionista ou participante, de forma direta ou indireta, recebimento por palestras, aulas, atuação como proctor de treinamentos, remunerações, honorários pagos por participações em conselhos consultivos, de investigadores, ou outros comitês, etc. Provenientes da indústria farmacêutica, de órteses, próteses, equipamentos e implantes, brasileiras ou estrangeiras: - Bayer: Nubeqa / Sankyo: Lixiana. Outros relacionamentos Financiamento de atividades de educação médica continuada, incluindo viagens, hospedagens e inscrições para congressos e cursos, provenientes da indústria farmacêutica, de órteses, próteses, equipamentos e implantes, brasileiras ou estrangeiras: - Boehringer: Jardiance Duo; Johnson's: Imbruvica. Atuação no último ano como auditor médico para empresa operadora de planos de saúde ou assemelhada: - Auditoria de prontuários: Lean Saúde.
Leticia Neves Solon Carvalho	Declaração financeira A - Pagamento de qualquer espécie e desde que economicamente apreciáveis, feitos a (i) você, (ii) ao seu cônjuge/ companheiro ou a qualquer outro membro que resida com você, (iii) a qualquer pessoa jurídica em que qualquer destes seja controlador, sócio, acionista ou participante, de forma direta ou indireta, recebimento por palestras, aulas, atuação como proctor de treinamentos, remunerações, honorários pagos por participações em conselhos consultivos, de investigadores, ou outros comitês, etc. Provenientes da indústria farmacêutica, de órteses, próteses, equipamentos e implantes, brasileiras ou estrangeiras: - Novartis: Inclisiran / Dislipidemia Outros relacionamentos Financiamento de atividades de educação médica continuada, incluindo viagens, hospedagens e inscrições para congressos e cursos, provenientes da indústria farmacêutica, de órteses, próteses, equipamentos e implantes, brasileiras ou estrangeiras: - Novartis: Inclisiran / Dislipidemia; Novonordisk: Semaglutida / Obesidade.
Luciana Dornfeld Bichuette	Nada a ser declarado
Luka David Lechinewski	Declaração financeira A - Pagamento de qualquer espécie e desde que economicamente apreciáveis, feitos a (i) você, (ii) ao seu cônjuge/ companheiro ou a qualquer outro membro que resida com você, (iii) a qualquer pessoa jurídica em que qualquer destes seja controlador, sócio, acionista ou participante, de forma direta ou indireta, recebimento por palestras, aulas, atuação como proctor de treinamentos, remunerações, honorários pagos por participações em conselhos consultivos, de investigadores, ou outros comitês, etc. Provenientes da indústria farmacêutica, de órteses, próteses, equipamentos e implantes, brasileiras ou estrangeiras: - Servier: Acertalix/Acertanlo/Acertil/Triplixam; Bayer: Firialta. Outros relacionamentos Financiamento de atividades de educação médica continuada, incluindo viagens, hospedagens e inscrições para congressos e cursos, provenientes da indústria farmacêutica, de órteses, próteses, equipamentos e implantes, brasileiras ou estrangeiras: - Chiesi: Trimbow; Servier: Acertalix/triplixam.
Marcelle Gonçalves Henriques Lizandro	Nada a ser declarado
Marcelo Bettega	Nada a ser declarado
Marco Antonio Freitas de Queiroz Mauricio Filho	Nada a ser declarado
Maria Antonieta Albanez Albuquerque de Medeiros Lopes	Declaração financeira A - Pagamento de qualquer espécie e desde que economicamente apreciáveis, feitos a (i) você, (ii) ao seu cônjuge/ companheiro ou a qualquer outro membro que resida com você, (iii) a qualquer pessoa jurídica em que qualquer destes seja controlador, sócio, acionista ou participante, de forma direta ou indireta, recebimento por palestras, aulas, atuação como proctor de treinamentos, remunerações, honorários pagos por participações em conselhos consultivos, de investigadores, ou outros comitês, etc. Provenientes da indústria farmacêutica, de órteses, próteses, equipamentos e implantes, brasileiras ou estrangeiras: - Medtronic: Speaker; Boston Scientific: Speaker. Outros relacionamentos Financiamento de atividades de educação médica continuada, incluindo viagens, hospedagens e inscrições para congressos e cursos, provenientes da indústria farmacêutica, de órteses, próteses, equipamentos e implantes, brasileiras ou estrangeiras: - Boston: viagem como speaker
Matheus de Oliveira Laterza Ribeiro	Nada a ser declarado
Pedro Felipe Gomes Nicz	Declaração financeira A - Pagamento de qualquer espécie e desde que economicamente apreciáveis, feitos a (i) você, (ii) ao seu cônjuge/ companheiro ou a qualquer outro membro que resida com você, (iii) a qualquer pessoa jurídica em que qualquer destes seja controlador, sócio, acionista ou participante, de forma direta ou indireta, recebimento por palestras, aulas, atuação como proctor de treinamentos, remunerações, honorários pagos por participações em conselhos consultivos, de investigadores, ou outros comitês, etc. Provenientes da indústria farmacêutica, de órteses, próteses, equipamentos e implantes, brasileiras ou estrangeiras: - Boston Scientific Corporation - IVUS / Rotablator; Edwards Lifesciences - TAVI; Medtronic - TAVI; Microport Scientific Corporation - TAVI. Outros relacionamentos Financiamento de atividades de educação médica continuada, incluindo viagens, hospedagens e inscrições para congressos e cursos, provenientes da indústria farmacêutica, de órteses, próteses, equipamentos e implantes, brasileiras ou estrangeiras: - Meril Life Sciences - Intervenção Estrutural
Pedro Henrique de Moraes Cellia	Declaração financeira A - Pagamento de qualquer espécie e desde que economicamente apreciáveis, feitos a (i) você, (ii) ao seu cônjuge/ companheiro ou a qualquer outro membro que resida com você, (iii) a qualquer pessoa jurídica em que qualquer destes seja controlador, sócio, acionista ou participante, de forma direta ou indireta, recebimento por palestras, aulas, atuação como proctor de treinamentos, remunerações, honorários pagos por participações em conselhos consultivos, de investigadores, ou outros comitês, etc. Provenientes da indústria farmacêutica, de órteses, próteses, equipamentos e implantes, brasileiras ou estrangeiras: - Biolab: evolucumab / dislipidemia; - Novo Nordisk: semaglutida / diabetes Outros relacionamentos Financiamento de atividades de educação médica continuada, incluindo viagens, hospedagens e inscrições para congressos e cursos, provenientes da indústria farmacêutica, de órteses, próteses, equipamentos e implantes, brasileiras ou estrangeiras: - Novo Nordisk: semaglutida Possui qualquer outro interesse (financeiro ou a qualquer outro título) que deva ser declarado tendo em vista o cargo ocupado na SBC, ainda que não expressamente elencado anteriormente: - Instituto Pulsare: serviços médicos
Sarah Fagundes Grobe	Declaração financeira A - Pagamento de qualquer espécie e desde que economicamente apreciáveis, feitos a (i) você, (ii) ao seu cônjuge/ companheiro ou a qualquer outro membro que resida com você, (iii) a qualquer pessoa jurídica em que qualquer destes seja controlador, sócio, acionista ou participante, de forma direta ou indireta, recebimento por palestras, aulas, atuação como proctor de treinamentos, remunerações, honorários pagos por participações em conselhos consultivos, de investigadores, ou outros comitês, etc. Provenientes da indústria farmacêutica, de órteses, próteses, equipamentos e implantes, brasileiras ou estrangeiras: - Biolab: Doble; Novartis: Sybrava; Ache: dislipidemia; Servier: Vastarel; Mantecorp: Saúde da mulher; Daiichi Sankyo: Nustenti. B - Financiamento de pesquisas sob sua responsabilidade direta/pessoal (direcionado ao departamento ou instituição) provenientes da indústria farmacêutica, de órteses, próteses, equipamentos e implantes, brasileiras ou estrangeiras: - Servier: Vastarel; Daiichi Sankyo: Nustendi. Outros relacionamentos Financiamento de atividades de educação médica continuada, incluindo viagens, hospedagens e inscrições para congressos e cursos, provenientes da indústria farmacêutica, de órteses, próteses, equipamentos e implantes, brasileiras ou estrangeiras: - Servier; Novartis.
Sayuri Inuzuka	Declaração financeira A - Pagamento de qualquer espécie e desde que economicamente apreciáveis, feitos a (i) você, (ii) ao seu cônjuge/ companheiro ou a qualquer outro membro que resida com você, (iii) a qualquer pessoa jurídica em que qualquer destes seja controlador, sócio, acionista ou participante, de forma direta ou indireta, recebimento por palestras, aulas, atuação como proctor de treinamentos, remunerações, honorários pagos por participações em conselhos consultivos, de investigadores, ou outros comitês, etc. Provenientes da indústria farmacêutica, de órteses, próteses, equipamentos e implantes, brasileiras ou estrangeiras: - Servier: Anti-hipertensivos / Biolab: Anti-hipertensivos Outros relacionamentos Financiamento de atividades de educação médica continuada, incluindo viagens, hospedagens e inscrições para congressos e cursos, provenientes da indústria farmacêutica, de órteses, próteses, equipamentos e implantes, brasileiras ou estrangeiras: - Servier: Anti-hipertensivos
Stefano Garzon Dias Lemos	Declaração financeira A - Pagamento de qualquer espécie e desde que economicamente apreciáveis, feitos a (i) você, (ii) ao seu cônjuge/ companheiro ou a qualquer outro membro que resida com você, (iii) a qualquer pessoa jurídica em que qualquer destes seja controlador, sócio, acionista ou participante, de forma direta ou indireta, recebimento por palestras, aulas, atuação como proctor de treinamentos, remunerações, honorários pagos por participações em conselhos consultivos, de investigadores, ou outros comitês, etc. Provenientes da indústria farmacêutica, de órteses, próteses, equipamentos e implantes, brasileiras ou estrangeiras: - Boston scientific: speaker e proctor; Abbott: speaker e proctor. B - Financiamento de pesquisas sob sua responsabilidade direta/pessoal (direcionado ao departamento ou instituição) provenientes da indústria farmacêutica, de órteses, próteses, equipamentos e implantes, brasileiras ou estrangeiras: - Amplifier: análise de imagem cardíaca em oclusão de apêndice atrial esquerdo.
Tannas Jatene	Nada a ser declarado
Wilton Francisco Gomes	Nada a ser declarado

## Sumário

**Resumo Executivo/Mensagens-chave** 7
**1. Apresentação do Posicionamento e Metodologia** 7
  **1.1. Metodologia** 7
  **1.2. Processo de Construção** 7
  **1.3. Natureza das Recomendações** 7
**2. Introdução** 9
**3. Acometimento Cardiovascular** 9
**4. Exames Complementares e Avaliação Prognóstica** 10
**5. Considerações no Manejo de Antitrombóticos e Anticoagulantes em Pacientes com Dengue** 11
**6. Manejo de Antiagregantes em Pacientes com Dengue** 12
  **6.1. Recomendações Específicas para o Manejo de Antiplaquetários em pacientes com Dengue e Plaquetopenia** 12
  **6.2. Angioplastia com Stent < 30 Dias** 12
  **6.3. Angioplastia com Stent ≥ 30 Dias** 13
  **6.4. Demais Cenários Clínicos sob Uso de Monoterapia (AAS ou P2Y12)** 13
  **6.5. Retorno da Terapia Antiplaquetária** 13
**7. Manejo da Anticoagulação em Pacientes com Plaquetopenia e Dengue** 13
  **7.1. Princípios Gerais** 13
  **7.2. Pacientes com Baixo Risco Trombótico** 13
  **7.3. Pacientes com Alto Risco Trombótico** 13
  **7.4. Retorno da Anticoagulação** 14
**8. Manejo de Sangramento Agudo** 14
  **8.1. Condutas Gerais** 14
  **8.2. Manejo em Usuários de Antiplaquetários** 14
  **8.3. Manejo do Sangramento em Usuários de Varfarina** 15
  **8.4. Manejo do Sangramento em Usuários de DOACs** 15
**9. Retorno das Terapias após Sangramento Agudo** 15
**10. Conclusão** 16
**Referências** 16

## Resumo executivo/Mensagens-chave

- A dengue apresenta elevada incidência no Brasil, com impacto sanitário e clínico relevante, especialmente em períodos epidêmicos;
- A dengue cursa com trombocitopenia, disfunção plaquetária e dano endotelial, elevando substancialmente o risco hemorrágico, sobretudo entre o 3º e 7º dia de doença;
- Todo paciente com plaquetas < 50.000/mm³ deve ser internado (Figura Central);
- Pacientes com angioplastia há < 30 dias constituem o grupo de maior risco trombótico, devendo-se priorizar a manutenção da dupla antiagregação plaquetária sempre que plaquetas ≥ 30.000/mm³;
- Para angioplastias ≥ 30 dias, a conduta é mais flexível, sendo possível manter apenas monoterapia com inibidor de P2Y12 sem ácido acetilsalicílico (AAS), quando as plaquetas estiverem entre 30.000 e 100.000/mm³;
- Em prevenção secundária em monoterapia (AAS ou P2Y12), manter o antiplaquetário se plaquetas ≥ 30.000/mm³; suspender quando < 30.000/mm³;
- Na anticoagulação oral, manter quando plaquetas ≥ 50.000/mm³ e suspender quando < 50.000/mm³;
- Pacientes com alto risco trombótico (prótese mecânica, fibrilação atrial [FA] com CHA_2_DS_2_-VASc ≥ 5, estenose mitral moderada/grave, trombo recente) devem ser acompanhados em ambiente de maior vigilância;
- Quando as plaquetas estiverem entre 30.000 e 50.000/mm³ em pacientes de alto risco trombótico, preferir heparina não fracionada em bomba, devido à meia-vida curta e à reversibilidade rápida
- O retorno dos antiplaquetários e anticoagulantes deve ocorrer na fase de recuperação, após estabilização clínica, ausência de sangramento e plaquetas acima dos limiares recomendados, podendo ser retomado de forma escalonada (Tabela 1).

**Tabela 1:** Resumo operacional

**1) Antiagregantes – Pacientes com *stent* < 30 dias**		
Plaquetas		Conduta
	≥ 30.000/mm³	Manter AAS + P2Y12 (preferir clopidogrel)
	< 30.000/mm³	Suspender ambos; considerar transfusão; monitorização intensiva
**2) Antiagregantes – *stent* ≥ 30 dias**		
Plaquetas		Conduta
	≥ 100.000/mm³	Manter DAPT
	30.000–99.999/mm³	Manter inibidor de P2Y12 e suspender AAS
	< 30.000/mm³	Suspender ambos; reiniciar de forma escalonada após estabilidade
**3) Demais contextos clínicos sob uso de monoterapia (AAS ou P2Y12)**		
Plaquetas		Conduta
	≥ 30.000/mm³	Manter monoterapia
	< 30.000/mm³	Suspender monoterapia até recuperação
**4) Anticoagulação – pacientes com baixo risco trombótico**		
Plaquetas		Conduta
	≥ 50.000/mm³	Manter anticoagulação
	< 50.000/mm³	Suspender anticoagulação; monitorização seriada
**5) Anticoagulação – pacientes com alto risco trombótico**		
Plaquetas		Conduta
	30.000–50.000/mm³	Preferir heparina não fracionada em bomba; monitorar TTPa
	< 30.000/mm³	Suspender anticoagulação; vigilância frequente; reintroduzir quando ≥ 50.000/mm³

AAS: ácido acetilsalicílico; DAPT: *dual antiplatelet therapy* (dupla antiagregação plaquetária); TTPa: tempo de tromboplastina parcial ativado.

## 1. Apresentação do Posicionamento e Metodologia

### 1.1. Metodologia

Este documento reúne evidências disponíveis com o objetivo de fornecer orientações práticas e padronizadas para o manejo de antiplaquetários e anticoagulantes em pacientes com dengue, especialmente na presença de plaquetopenia e risco hemorrágico aumentado.

### 1.2. Processo de Construção

1. Revisão narrativa da literatura, com buscas estruturadas nas principais bases biomédicas (PubMed, Embase e Cochrane), incluindo estudos observacionais, séries e relatos de casos, revisões sistemáticas e documentos oficiais do Ministério da Saúde;
2. Avaliação de evidências indiretas, provenientes de diretrizes internacionais e de recomendações aplicáveis ao manejo de plaquetopenia em outras etiologias;
3. Discussão estruturada e consenso entre especialistas, conforme prática habitual em posicionamentos da Sociedade Brasileira de Cardiologia.

### 1.3. Natureza das Recomendações

As recomendações apresentadas são baseadas, predominantemente, em opinião de especialistas, apoiada por evidências indiretas, uma vez que não existem ensaios clínicos randomizados dedicados ao manejo de antiplaquetários ou anticoagulantes em pacientes com dengue. Portanto, as decisões clínicas devem ser individualizadas, considerando o equilíbrio entre risco hemorrágico e risco trombótico.

**Figure:**
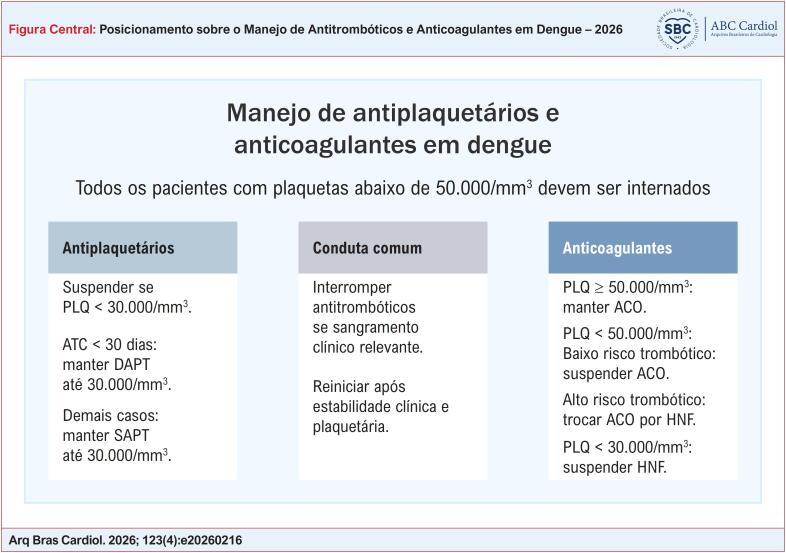
ACO: anticoagulante oral; ATC: angioplastia percutânea; DAPT: dupla antiagregação plaquetária; HNF: heparina não fracionada; PLQ: plaquetas; SAPT: monoterapia antiplaquetária.

## 2. Introdução

A dengue é uma arbovirose causada pelo vírus da dengue (DENV), do gênero flavivírus, com quatro sorotipos (DENV-1 a DENV-4).^1,2^ Nas últimas 5 décadas, observou-se crescimento expressivo da incidência global, estimando-se mais de 500 milhões de infecções anuais, das quais cerca de 96 milhões eram sintomáticas.^3,4^ A Organização Mundial da Saúde (OMS) classifica a dengue como uma das arboviroses de maior relevância em saúde pública.

No Brasil, a doença é endêmica e apresentou, em 2024, o maior surto já registrado na América Latina, com aumento significativo de casos em relação a 2023, segundo a Organização Pan-Americana da Saúde.^5^ A dengue é de notificação compulsória desde 1961, e epidemias recorrentes têm sido descritas desde 1986. Entre 2008 e 2019, mais de 6.000 óbitos foram atribuídos à doença.^6^ A expansão recente está associada à disseminação simultânea dos quatro sorotipos, resistência vetorial a inseticidas, urbanização rápida, saneamento insuficiente e mudanças climáticas.^6^

A apresentação clínica varia amplamente: a maioria dos casos é assintomática ou leve, mas cerca de 5% dos pacientes hospitalizados evoluem com formas graves.^7^ A infecção evolui em três fases: febril, crítica e de recuperação.^8^

A fase febril dura de 2 a 7 dias e caracteriza-se por febre abrupta (39–40 °C), cefaleia, dor retro-orbitária, mialgia, artralgia, sintomas gastrointestinais e exantema em até 50% dos casos.

A fase crítica, entre o 3º e 7º dia, inicia-se geralmente na defervescência e caracteriza-se por aumento da permeabilidade capilar, hemoconcentração e risco hemorrágico e de choque. Nessa fase, podem ocorrer taquicardia, bradicardia, arritmias, distúrbios de condução atrioventricular, miocardite e, nos casos mais graves, choque circulatório decorrente de extravasamento plasmático.^8^ A OMS recomenda o termo "dengue grave" para essas situações, reservando "dengue grave com fenômenos hemorrágicos" quando sangramentos relevantes estão presentes.^8^

Os sinais de alarme incluem dor abdominal intensa, vômitos persistentes, derrames cavitários, hipotensão postural, hepatomegalia dolorosa, sangramentos de mucosas, letargia/irritabilidade e aumento do hematócrito.

A dengue grave é definida pela presença de choque, desconforto respiratório por congestão pulmonar, sangramento grave ou disfunção orgânica importante – hepática, neurológica (como convulsões, encefalite ou coma), cardíaca (miocardite, insuficiência cardíaca, choque cardiogênico) ou renal (insuficiência aguda).

Na fase de recuperação, há reabsorção do líquido extravasado, melhora clínica progressiva, normalização hemodinâmica e recuperação tardia da contagem de plaquetas. Um exantema tardio pode surgir, e adultos frequentemente apresentam astenia prolongada."

A seguir, exploramos maior detalhamento fisiopatológico quanto às distintas formas de acometido cardíaco pela dengue.

## 3. Acometimento Cardiovascular

**Figura 1:**
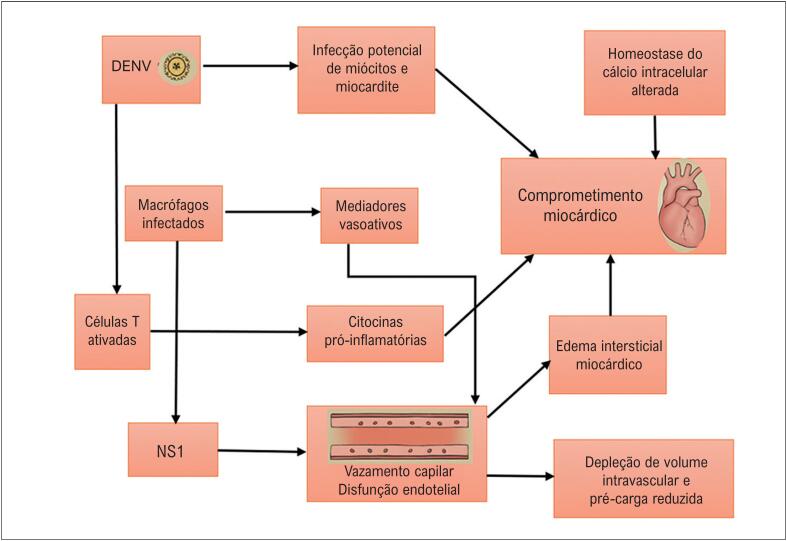
Mecanismos virais e imunes envolvidos no acometimento cardiovascular da dengue. DENV: dengue vírus; NS1: proteína não estrutural 1. Fonte: Adaptado de Yacoub et al.^12^

O acometimento cardiovascular pelo DENV é amplo e não se limita ao miocárdio, podendo envolver o sistema de condução, estruturas vasculares, sistema autonômico, interstício e o pericárdio.^9^ A miocardite é reconhecida pela OMS como manifestação de dengue grave desde 2009, com incidência estimada em 12,4%,^10^ enquanto formas cardiovasculares autolimitadas, que regridem na fase de recuperação, ocorrem em aproximadamente 15 a 20% dos casos graves (Figura 1).^11^

A disfunção miocárdica pode resultar de efeitos diretos do vírus, mas também de mecanismos indiretos, como edema miocárdico por extravasamento capilar, ação de mediadores inflamatórios circulantes, hipoperfusão coronária e alterações da homeostase do cálcio.^11,12^ A pericardite, quando presente, é geralmente concomitante à miocardite, sustentada pelo extravasamento capilar e sem grandes volumes de derrame; em uma coorte prospectiva cubana de 427 pacientes, sua incidência foi de 1,6%.^13^

Além dos efeitos diretos no coração, a infecção por dengue parece associar-se ao aumento do risco de eventos isquêmicos agudos. Em um estudo com mais de 65 mil pacientes, observou-se aumento do risco de infarto agudo do miocárdio e acidente vascular cerebral nas duas primeiras semanas após a infecção.^14^

A trombocitopenia e o sangramento têm fisiopatologia complexa e multifatorial. Na fase febril, há ativação endotelial e hemostática, com elevação de marcadores como antígeno do fator de von Willebrand e o inibidor do ativador de plasminogênio tipo 1 (PAI-1), concomitante à queda progressiva das plaquetas.^15^ A redução da produção medular de plaquetas decorre, em parte, da ação viral direta sobre células progenitoras hematopoéticas.^16^

A proteína não estrutural 1 (NS1) ativa plaquetas via receptor Toll-like 4 (TLR4), contribuindo para trombocitopenia e sangramento,^17^ enquanto a ativação do inflamassoma NLRP3 induz a liberação de interleucina (IL)-1β, aumentando a permeabilidade endotelial.^18^ Na fase crítica, níveis elevados de citocinas inflamatórias, como fator de necrose tumoral-α (TNF-α), IL-6, IL-13 e IL-18, correlacionam-se com maior permeabilidade capilar e risco hemorrágico.^16,19^

A NS1 e o próprio vírus também aumentam a atividade da heparanase, levando à degradação do heparan sulfato, componente-chave do glicocálice endotelial.^12^ A perda dessa barreira compromete a integridade das junções intercelulares e favorece o extravasamento plasmático.^20^ A trombocitopenia é, ainda, agravada pela ativação plaquetária mediada por IL-6 e indução do fator tecidual, que impulsiona a coagulação sistêmica.^16^

Esses mecanismos demonstram que a combinação entre disfunção plaquetária, permeabilidade capilar aumentada e resposta inflamatória exacerbada constitui o núcleo fisiopatológico da trombocitopenia e do sangramento na dengue. Essa condição representa um desafio particular para o manejo de pacientes cardiopatas que necessitam de antiplaquetários ou anticoagulantes, devido ao impacto direto desses fármacos no risco hemorrágico.

## 4. Exames Complementares e Avaliação Prognóstica

Na avaliação de pacientes com suspeita de dengue, alguns exames auxiliam não apenas na confirmação diagnóstica, mas também na identificação precoce daqueles com maior risco de evoluir para formas graves.

O diagnóstico laboratorial pode ser realizado por métodos diretos, como isolamento viral ou transcriptase reversa seguida de reação em cadeia da polimerase (RT-PCR), e por métodos indiretos, como detecção de anticorpos da imunoglobulina M (IgM) por meio do ensaio imunoenzimático (ELISA), pesquisa do antígeno NS1, teste de inibição da hemaglutinação ou neutralização por redução de placas.^21^ A escolha do método e o momento adequado para a coleta devem seguir as orientações da vigilância epidemiológica local.

Como a resposta imunológica da dengue não permite identificar a infecção aguda apenas pela presença de anticorpos, o teste rápido para detecção do antígeno NS1 é o mais indicado nesse período.^21^ Trata-se de uma ferramenta útil para decisões clínicas rápidas e individualizadas.^22^

Entre os exames de rotina, destacam-se as alterações no hemograma. Plaquetopenia acentuada, linfocitopenia e leucopenia estão associadas a maior risco de sangramento e falência orgânica, sendo observadas em aproximadamente 70%, 67% e 68,3% dos pacientes com dengue grave, respectivamente.^23^

A Tabela 2 resume os exames recomendados na suspeita clínica de dengue, enquanto a Tabela 3 apresenta as principais alterações laboratoriais associadas à forma grave da doença.

**Tabela 2:** Exames na suspeita clínica de dengue

Categoria	Exames recomendados	Finalidade clínica-científica
Hemograma	Hemograma completo	Identificar leucopenia, trombocitopenia e hemoconcentração
Função hepática	AST (TGO), ALT (TGP), bilirrubinas, albumina	Avaliar possível acometimento hepático associado a dengue
Coagulação	TAP (RNI), TTPa, fibrinogênio	Detectar alterações de coagulação e risco hemorrágico
Função renal e eletrólitos	Ureia, creatinina, sódio, potássio	Avaliar complicações metabólicas e renais
Marcadores específicos	NS1, sorologia IgM/IgG, RT-PCR	Confirmação diagnóstica do vírus da dengue
Exames complementares	Gasometria, proteína C reativa	Avaliar gravidade, resposta inflamatória e necessidade de suporte adicional

ALT (TGP): alanina aminotransferase (transaminase glutâmico-pirúvica); AST (TGO): aspartato aminotransferase (transaminase glutâmico-oxalacética); IgM/IgG: imunoglobulina M/imunoglobulina G; NS1: proteína não estrutural 1 vírus da dengue; RT-PCR: transcriptase reversa seguida de reação em cadeia da polimerase; TAP (RNI): tempo de atividade de protrombina (razão normalizada internacional); TTPa: tempo de tromboplastina parcial ativado.

**Tabela 3:** Principais alterações laboratoriais associadas à dengue grave

Exame/Categoria	Alteração/Achado laboratorial	Frequência (%)	Significado clínico e prognóstico
Hemograma	Trombocitopenia(< 100.000/mm³; < 50.000/mm³)	70–81,9;^24^ 42,6^25^	Forte associação com progressão para formas graves e risco de sangramento
Hemograma	Leucopenia/Linfocitopenia	68,3;^23^ 22,2–67,2^23-25^	Achado comum; leucocitose pode ocorrer em adultos com doença grave
Hemograma	Monocitose/Linfocitose atípica	67^23^	Resposta imunológica viral; achado inespecífico
Hemograma	Contagem normal de plaquetas com manifestações hemorrágicas	-	Sugere disfunção plaquetária
Hematócrito	Elevação significativa em relação ao basal	-	Sinal clássico de hemoconcentração; indica risco aumentado de choque
Hematócrito	Hematócrito baixo	-	Sugere sangramento agudo
Função hepática	Transaminases (AST/ALT) elevadas (> 400 U/L, pico 4º–6º dia)	AST 81,2–84,9%; ALT 52,5–73,3^25-26^	Indica envolvimento hepático; associada a maior risco de dengue grave
Função hepática	Hipoalbuminemia (< 3,5 g/L)	52,9^25^	Reflete extravasamento plasmático; marcador independente de gravidade
Coagulação	TTPa aumentado	42,9^24^	Sugere coagulopatia e risco hemorrágico
Coagulação	TP prolongado (RNI elevado)	16,5^24^	Correlaciona-se com disfunção hepática e risco de sangramento
Coagulação	Fibrinogênio reduzido/Dímero D discretamente elevado	-	Indicador de fibrinólise e gravidade
Coagulação	Coombs indireto positivo	-	Raro; indica hemólise imunológica
Função renal e eletrólitos	Ureia, creatinina, sódio, potássio	-	Avaliar complicações renais e distúrbios metabólicos
Exames complementares	Gasometria, proteína C reativa	-	Avaliação da gravidade e resposta inflamatória
Marcadores específicos	NS1, sorologia IgM/IgG, RT-PCR	-	Confirmação diagnóstica da dengue

ALT: alanina aminotransferase; AST: aspartato aminotransferase; IgM/IgG: imunoglobulina M/imunoglobulina G; RNI: razão normalizada internacional; NS1: proteína não estrutural 1 do vírus da dengue; RT-PCR: transcriptase reversa seguida de reação em cadeia da polimerase; TP: tempo de protrombina; TTPa: tempo de tromboplastina parcial ativado.

## 5. Considerações no Manejo de Antitrombóticos e Anticoagulantes em Pacientes com Dengue

As recomendações atuais são baseadas em dados provenientes de relatos de casos,^27^ séries de casos,^28^ diretrizes e documentos nacionais^7,15^ e no consenso dos autores desse posicionamento. Também foram consideradas evidências indiretas no manejo de antitrombóticos e anticoagulação em pacientes com plaquetopenia por outras causas, como, por exemplo, as neoplasias.^29-31^

De modo geral, recomenda-se a manutenção de anticoagulantes e antiplaquetários no contexto neoplásico com plaquetas acima de 50.000/mm^3,30^ sendo que a nova diretriz de síndrome coronariana crônica orienta a possibilidade de manutenção de dupla antiagregação plaquetária (DAPT) em pacientes oncológicos com valores plaquetários acima de 30.000/mm^3^.^31^

No entanto, visto que, na fisiopatologia da dengue, há cenários particulares como inflamação, disfunção plaquetária e endotelial que alteram o risco hemorrágico, serão feitas orientações particulares a respeito do manejo desses pacientes nos parágrafos abaixo.

## 6. Manejo de Antiagregantes em Pacientes com Dengue

A elevada incidência de dengue no Brasil, frequentemente marcada por epidemias anuais, impõe desafios relevantes ao manejo de pacientes cardiopatas em uso de antiplaquetários ou anticoagulantes.^32^ A trombocitopenia típica da doença, somada à disfunção endotelial e às alterações da coagulação, aumenta substancialmente o risco de sangramentos, tornando o uso de fármacos que interferem na hemostasia um ponto crítico da tomada de decisão clínica.^33^

Nesse cenário, torna-se essencial avaliar cuidadosamente o risco-benefício da continuidade ou suspensão da terapia antitrombótica, equilibrando o risco de sangramento com o potencial risco trombótico decorrente da interrupção do tratamento. A contagem de plaquetas, o risco trombótico individual e o contexto clínico do uso dos antiagregantes constituem parâmetros centrais para orientar condutas.

A doença apresenta amplo espectro clínico, desde quadros febris autolimitados, até formas graves com sangramento, disfunções orgânicas e choque. A trombocitopenia com disfunção plaquetária é marcante, e o maior risco de sangramento concentra-se na transição entre o final da febre e os primeiros dias da fase crítica, tipicamente entre o 3º e 5º dias e até 2 dias após o desaparecimento da febre.^34^

Pacientes submetidos à angioplastia com *stent* requerem DAPT por períodos definidos conforme o tipo de *stent* e a condição clínica.^35,36^ A suspensão de antiplaquetários – frequentemente necessária diante da plaquetopenia associada à dengue – pode aumentar o risco de trombose de *stent*, evento grave que usualmente se manifesta como infarto do miocárdio e apresenta alta mortalidade.^37,38^ Estudos apontam que a interrupção precoce da DAPT é o principal preditor de trombose do *stent*, com o período de maior vulnerabilidade concentrado nos primeiros 30 dias após o implante.^39,40^

### 6.1. Recomendações Específicas para o Manejo de Antiplaquetários em Pacientes com Dengue e Plaquetopenia

Recomendamos que todos os pacientes com nível plaquetário inferior a 50.000/mm^3^ plaquetas devem ser internados para avaliação clínica e laboratorial.

O manejo em relação à manutenção ou suspensão de antiplaquetários encontra-se nos tópicos abaixo.

### 6.2. Angioplastia com *Stent* < 30 Dias

Pacientes desse grupo apresentam muito alto risco trombótico,^41,42^ exigindo cautela na suspensão da terapia antiplaquetária:

- Plaquetas ≥ 30.000/mm³: recomenda-se manter AAS + P2Y12, com preferência ao clopidogrel;^43-48^
- Plaquetas < 30.000/mm³: recomenda-se suspender ambos;
- Pode-se considerar transfusão de plaquetas < 30.000/mm³ para manutenção de monoterapia, em especial em pacientes mais sintomáticos.^30^

### 6.3. Angioplastia com *Stent* ≥ 30 Dias

- Plaquetas ≥ 100.000/mm³: recomenda-se manter DAPT;
- Plaquetas 30.000 a 99.999/mm³: recomenda-se manter P2Y12 e suspender AAS;
- Plaquetas < 30.000/mm³: recomenda-se suspender ambos.

As recomendações acima também podem ser aplicadas a pacientes com infarto agudo do miocárdio recente sob tratamento clínico ou revascularização cirúrgica.

Pacientes com alto risco isquêmico (por exemplo: múltiplos *stents*, lesões longas, tronco da coronária esquerda, bifurcações verdadeiras, diabetes mellitus ou doença renal crônica) podem requerer individualização, sendo possível manter DAPT com plaquetas ≥ 50.000/mm³.

### 6.4. Demais Cenários Clínicos sob Uso de Monoterapia (AAS ou P2Y12)

Inclui pacientes em prevenção secundária (pacientes em tratamento clínico, pós-revascularização miocárdica cirúrgica ou que tenham completado o tempo recomendado de DAPT):

- Plaquetas ≥ 30.000/mm³: recomenda-se manter;
- Plaquetas < 30.000/mm³: recomenda-se suspender.

### 6.5. Retorno da Terapia Antiplaquetária

- Recomenda-se retomada somente na fase de recuperação, sem evidência de sangramento e com plaquetas acima dos limiares definidos;
- Pode-se considerar a retomada gradual, iniciando por monoterapia com o inibidor de P2Y12 e posterior retomada de aspirina naqueles pacientes com indicação clínica.

## 7. Manejo da Anticoagulação em Pacientes com Plaquetopenia e Dengue

A decisão deve ser individualizada, ponderando o risco de sangramento e o risco trombótico de forma criteriosa.

A avaliação do risco de sangramento deve considerar:

- Gravidade da dengue;
- Histórico de sangramento moderado ou grave nos últimos 3 meses;
- Doença renal crônica (estágio ≥ III);
- Hepatopatia crônica moderada ou grave;
- Câncer ativo;
- Idade > 75 anos.

A plaquetopenia é frequente nos quadros graves de dengue. Estudos demonstram que plaquetas < 100.000/mm³ já se associam a maior risco de sangramento.^41,42^

Na avaliação do risco trombótico, considera-se com alto risco trombótico os pacientes:^43^

- Com próteses valvares mecânicas, especialmente em posição mitral;
- Com eventos tromboembólicos recentes (últimos 3 meses);
- Com FA e CHA2DS2-VASc ≥ 5;
- Com FA associada à estenose mitral moderada ou grave;
- Com trombo recente no ventrículo esquerdo (últimos 3 meses);
- Portadores de síndrome do anticorpo antifosfolípide.

A decisão sobre suspender ou ajustar a anticoagulação deve integrar o risco trombótico, o grau de plaquetopenia, o tipo de anticoagulante e as condições clínicas do paciente.

Importante: em pacientes com próteses mecânicas ou FA associada à estenose mitral moderada/acentuada, o uso de anticoagulantes orais diretos (DOACs) é contraindicado. Nesses casos, deve-se utilizar cumarínicos (varfarina).^49-53^

### 7.1. Princípios Gerais

- Recomenda-se manter a anticoagulação quando plaquetas ≥ 50.000/mm³, na ausência de sangramento;
- Recomenda-se suspender a anticoagulação quando plaquetas < 50.000/mm³, com monitorização diária em ambiente hospitalar;
- Não se recomenda o uso de DOACs em próteses valvares mecânicas ou estenose mitral moderada/acentuada;
- Recomenda-se monitorar tempo de atividade de protrombina/razão normalizada internacional (TAP/RNI) em pacientes que utilizam varfarina;
- Recomenda-se classificar risco trombótico como alto quando houver:
  – Prótese valvar mecânica (especialmente mitral);
  – FA com CHA_2_DS_2_-VASc ≥ 5;
  – FA com estenose mitral moderada/grave;
  – Trombo ventricular esquerdo recente;
  – Síndrome antifosfolípide;
  – Evento tromboembólico nos últimos 3 meses.

### 7.2. Pacientes com Baixo Risco Trombótico

- Plaquetas < 50.000/mm³: recomenda-se suspender anticoagulação e internar;
- Recomenda-se reiniciar anticoagulação quando plaquetas ≥ 50.000/mm³ e ausência de sangramento.

### 7.3. Pacientes com Alto Risco Trombótico

- Plaquetas 30.000 a 50.000/mm³: recomenda-se substituir o anticoagulante oral por heparina não fracionada (HNF) em bomba de infusão:
  – A HNF deve ser preferida pela meia-vida curta e reversibilidade com protamina;
  – Recomenda-se monitorização seriada do tempo de tromboplastina parcial ativado (TTPa).
- Plaquetas < 30.000/mm³: recomenda-se suspender totalmente a anticoagulação.
- Para pacientes em varfarina:
  – Recomenda-se iniciar HNF quando RNI < 2,0.
- Para pacientes em DOACs:
  – Recomenda-se iniciar HNF 24 a 48 h após a última dose, considerando *clearance* renal.

### 7.4. Retorno da Anticoagulação

- Recomenda-se reiniciar anticoagulação somente na fase de recuperação, com:
  – Estabilização clínica;
  – Ausência de sangramento;
  – Plaquetas ≥ 50.000/mm³.
- Em pacientes em varfarina:
  – Recomenda-se considerar ponte com heparina de baixo peso molecular (HBPM) ou HNF até atingir RNI terapêutico.
- Podem-se considerar testes viscoelásticos (ROTEM delta/ROTEM platelet) em casos complexos.

**Figura 2:**
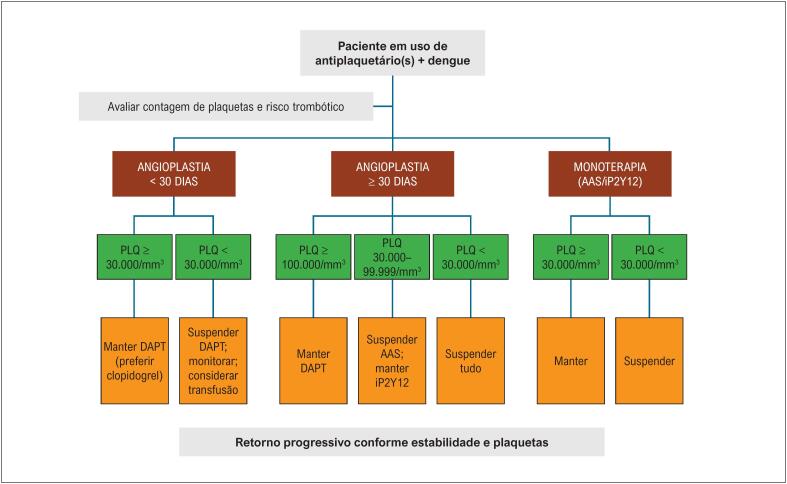
Manejo de antiplaquetários em pacientes com dengue. AAS: ácido acetilsalicílico; DAPT: dupla antiagregação antiplaquetária; iP2Y12: inibidor de P2Y12; PLQ: plaquetas.

As Figuras 2 e 3 resumem o manejo de antitrombóticos e anticoagulantes nos diferentes cenários clínicos.

**Figura 3:**
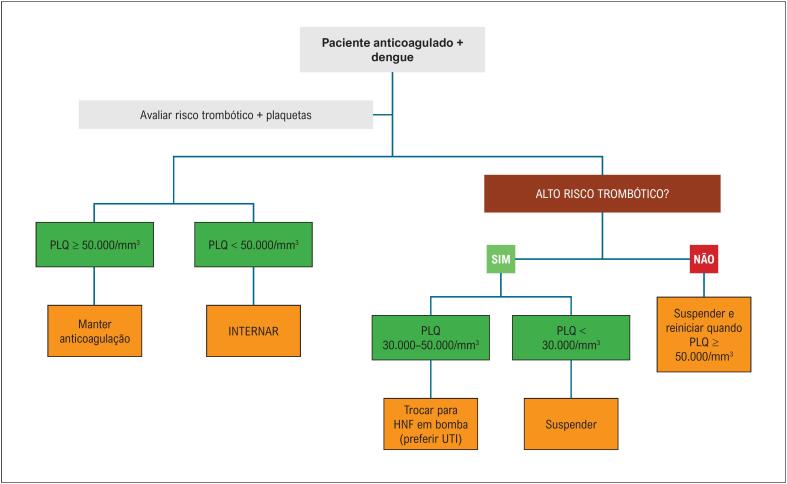
Manejo de anticoagulantes em pacientes com dengue. HNF: heparina não fracionada PLQ: plaquetas; UTI: unidade de terapia intensiva.

## 8. Manejo de Sangramento Agudo

### 8.1. Condutas Gerais

Recomenda-se suspender imediatamente todos os antiplaquetários e anticoagulantes diante de sangramento moderado ou grave;

- Recomenda-se internar o paciente em unidade de terapia intensiva (UTI) ou unidade de alta vigilância;
- Recomenda-se monitorar diariamente: plaquetas, hemoglobina/hematócrito, TAP/RNI, TTPa, função renal e hepática;
- Recomenda-se corrigir fatores agravantes (acidose, hipotermia, distúrbios eletrolíticos).

### 8.2. Manejo em Usuários de Antiplaquetários

- Em pacientes em DAPT, recomenda-se transfusão de plaquetas:
  – 1 unidade/10 kg, ou
  – 1 a 2 concentrados por aférese.
- Recomenda-se repetir transfusão se houver persistência de sangramento ou plaquetopenia crítica;
- Pode-se considerar monitorização contínua de eletrocardiograma em pacientes com *stent* recente.

### 8.3. Manejo do Sangramento em Usuários de Varfarina

- Recomenda-se utilizar concentrado de complexo protrombínico (PCC) para reversão imediata;
- Se PCC indisponível, recomenda-se plasma fresco congelado (PFC) (15 mL/kg) até RNI < 1,5;
- Recomenda-se administrar vitamina K 10 mg (via oral ou endovenosa);
- Recomenda-se monitorizar RNI seriado a cada 6 a 12 h.

### 8.4. Manejo do Sangramento em Usuários de DOACs

Dabigatrana:

- Recomenda-se idarucizumabe como antídoto específico;
- Na ausência, pode-se considerar PCC ativado (PCCa).

Inibidores do fator Xa (rivaroxabana, apixabana, edoxabana):

- Recomenda-se andexanet alfa;
- Se indisponível, recomenda-se PCC 4F.

Recomendações adicionais:

- Recomenda-se monitorização rigorosa de diurese, sinais vitais e perfusão;
- Recomenda-se evitar anti-inflamatórios não esteroidais;
- Pode-se considerar ácido tranexâmico em sangramento grave, se sem contraindicação.

**Figura 4:**
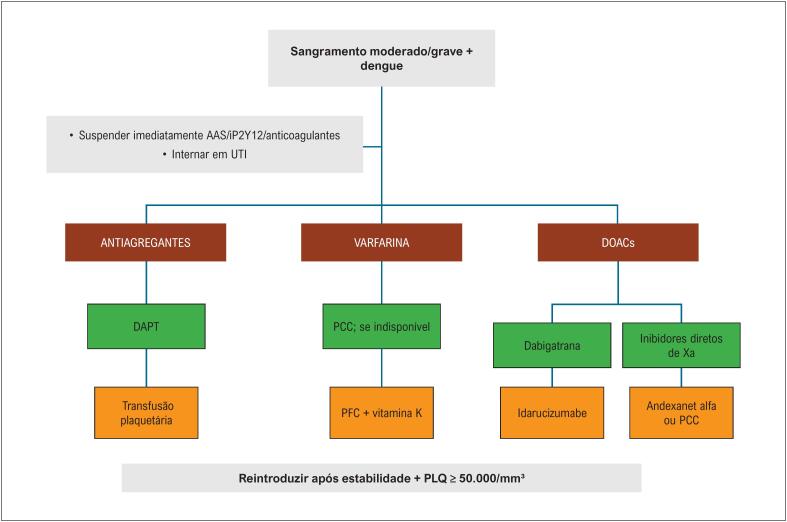
Manejo do sangramento em pacientes com dengue. AAS: ácido acetilsalicílico; DAPT: dupla antiagregação antiplaquetária; iP2Y12: inibidor de P2Y12; PLQ: plaquetas; PCC: complexo protrombínico; PFC: plasma fresco congelado; UTI: unidade de terapia intensiva.

A Figura 4 a seguir ilustra as condutas acima discutidas.

## 9. Retorno das Terapias após Sangramento Agudo

A transição da varfarina durante a dengue é particularmente desafiadora, pois a normalização do RNI após suspensão ou retomada ocorre de forma lenta, frequentemente exigindo ponte com HBPM até que o RNI retorne à faixa terapêutica. Embora a contagem de plaquetas continue sendo o critério mais utilizado para orientar o manejo da anticoagulação em pacientes com dengue, novas ferramentas vêm sendo estudadas para aprimorar a tomada de decisão. Entre elas, destacam-se os testes viscoelásticos, como o ROTEM platelet e o ROTEM delta, que oferecem avaliação integrada da função plaquetária, dos fatores de coagulação e da fibrinólise, permitindo detectar alterações não identificáveis por exames convencionais como o coagulograma.^54,55^

O uso seletivo dessas ferramentas pode beneficiar subgrupos de alto risco trombótico que ainda apresentam risco hemorrágico significativo, incluindo indivíduos com sangramentos de difícil controle, necessidade de cuidados intensivos, procedimentos invasivos recentes ou disfunção hepática grave.^56,57^ Diante disso, torna-se evidente a necessidade de critérios mais objetivos e validados para orientar a reintrodução segura da anticoagulação, ultrapassando o atual limiar empírico baseado apenas na contagem de plaquetas (> 50.000 a 75.000/mm³). A integração entre dados clínicos, exames laboratoriais tradicionais e, quando disponíveis, parâmetros viscoelásticos poderá, no futuro, permitir uma abordagem mais precisa, individualizada e segura nesses cenários complexos.

## 10. Conclusão

O aumento da incidência de dengue, com epidemias recorrentes, impõe desafios significativos no manejo clínico de pacientes em uso de antitrombóticos e anticoagulantes. O tratamento requer uma abordagem cuidadosa, equilibrando os riscos trombóticos e hemorrágicos. A interrupção temporária de terapias anticoagulantes ou antiplaquetárias pode ser necessária, mas deve ser individualizada, considerando a gravidade da infecção e as características clínicas do paciente.

## Data Availability

os conteúdos subjacentes ao texto do Posicionamento estão contidos no manuscrito.

## Lista de abreviaturas e siglas

AAS: Ácido acetilsalicílico (*aspirina*)
ALT (TGP): Alanina aminotransferase (transaminase glutâmico-pirúvica)
AST (TGO): Aspartato aminotransferase (transaminase glutâmico-oxalacética)
ATC: Angioplastia percutânea
CHA_2_DS_2_-VASc: Congestive Heart Failure, Hypertension, *Age ≥75, Diabetes*, *Stroke, Vascular disease*, *Age 65–74,Sex category (female)* – Escore de risco tromboembólico na fibrilação atrial
DAPT: *Dual antiplatelet therapy* (dupla antiagregação plaquetária)
DENV: *Dengue virus* – vírus da dengue
DENV-1 a DENV-4: Sorotipos 1 a 4 do vírus da dengue
DOAC: *Direct oral anticoagulant* – anticoagulante oral direto
ELISA: ensaio imunoenzimático (*enzyme-linked immunosorbent assay*)
FA: Fibrilação atrial
HBPM: Heparina de baixo peso molecular
HNF: Heparina não fracionada
IL: Interleucina
IL-6, IL-13, IL-18: Subtipos de interleucinas (citocinas pró-inflamatórias)
IgM/IgG: Imunoglobulina M/Imunoglobulina G
NLRP3: *NOD-like receptor family, pyrin domain containing 3* (inflamassoma NLRP3)
NS1: Proteína não estrutural 1 do vírus da dengue
OMS: Organização Mundial da Saúde
P2Y12: Receptor plaquetário de ADP (antagonista usado como antiagregante)
PAI-1: Inibidor do ativador de plasminogênio tipo 1
PCC: Concentrado de complexo protrombínico
PCCa: Concentrado de complexo protrombínico ativado
PFC: Plasma fresco congelado
PLQ: Plaquetas
RNI: Razão normalizada internacional (*international normalized ratio*)
ROTEM: *Rotational thromboelastometry* – tromboelastometria rotacional
ROTEM delta: Equipamento de tromboelastometria avançada
ROTEM platelet: Teste viscoelástico específico para função plaquetária
RT-PCR: Transcriptase reversa seguida de reação em cadeia da polimerase
SAPT: Monoterapia antiplaquetária
TAP: Tempo de atividade de protrombina
TLR4: Receptor Toll-like 4
TNF-α: Fator de necrose tumoral alfa
TTPa: Tempo de tromboplastina parcial ativado
UTI: Unidade de terapia intensiva

## References

[1] 1 Roy SK, Bhattacharjee S. Dengue Virus: Epidemiology, Biology, and Disease Aetiology. Can J Microbiol. 2021;67(10):687-702. doi: 10.1139/cjm-2020-0572. 10.1139/cjm-2020-0572 34171205

[2] 2 Kothari D, Patel N, Bishoyi AK. Dengue: Epidemiology, Diagnosis Methods, Treatment Options, and Prevention Strategies. Arch Virol. 2025;170(3):48. doi: 10.1007/s00705-025-06235-3. 10.1007/s00705-025-06235-3 39915348

[3] 3 Anumanthan G, Sahay B, Mergia A. Current Dengue Virus Vaccine Developments and Future Directions. Viruses. 2025;17(2):212. doi: 10.3390/v17020212. 10.3390/v17020212 PMC11861685 40006967

[4] 4 Pourzangiabadi M, Najafi H, Fallah A, Goudarzi A, Pouladi I. Dengue Virus: Etiology, Epidemiology, Pathobiology, and Developments in Diagnosis and Control - A Comprehensive Review. Infect Genet Evol. 2025;127:105710. doi: 10.1016/j.meegid.2024.105710. 10.1016/j.meegid.2024.105710 39732271

[5] 5 Ly H. Dengue Fever in the Americas. Virulence. 2024;15(1):2375551. doi: 10.1080/21505594.2024.2375551. 10.1080/21505594.2024.2375551 PMC11244333 38989831

[6] 6 Gurgel-Gonçalves R, Oliveira WK, Croda J. The Greatest Dengue Epidemic in Brazil: Surveillance, Prevention, and Control. Rev Soc Bras Med Trop. 2024;57:e002032024. doi: 10.1590/0037-8682-0113-2024. 10.1590/0037-8682-0113-2024 PMC11415067 39319953

[7] 7 Araiza-Garaygordobil D, García-Martínez CE, Burgos LM, Saldarriaga C, Liblik K, Mendoza I, et al. Dengue and the Heart. Cardiovasc J Afr. 2021;32(5):276-83. doi: 10.5830/CVJA-2021-033. 10.5830/CVJA-2021-033 PMC8756038 34292294

[8] 8 Brasil. Ministério da Saúde. Dengue - Diagnóstico e Manejo Clínico: Adulto e Criança. Brasília: Ministério da Saúde; 2024.

[9] 9 Teixeira MCR, Soares LFG, Pereira TR, Andrade BLM, Morais MO, Ferreira LM, et al. Explorando a Associação entre Dengue e seu Impacto Cardiovascular: Implicações Clínicas e Epidemiológicas. Braz J Health Rev. 2024;7(3): e69641. doi:10.34119/bjhrv7n3-081.

[10] 10 Farrukh AM, Ganipineni VDP, Jindal U, Chaudhary A, Puar RK, Ghazarian K, et al. Unveiling the Dual Threat: Myocarditis in the Spectrum of Dengue Fever. Curr Probl Cardiol;49(1): 102029. doi: 10.1016/j.cpcardiol.2023.102029.2024. 10.1016/j.cpcardiol.2023.102029 37567490

[11] 11 Cristodulo R, Luoma-Overstreet G, Leite F, Vaca M, Navia M, Durán G, et al. Dengue Myocarditis: A Case Report and Major Review. Glob Heart. 2023;18(1):41. doi: 10.5334/gh.1254. 10.5334/gh.1254 PMC10402786 37547170

[12] 12 Yacoub S, Wertheim H, Simmons CP, Screaton G, Wills B. Cardiovascular Manifestations of the Emerging Dengue Pandemic. Nat Rev Cardiol. 2014;11(6):335-45. doi: 10.1038/nrcardio.2014.40. 10.1038/nrcardio.2014.40 24710495

[13] 13 Cabrera-Rego JO, Rojas-Quiroz AF, Vidal-Turruelles Y, Yanes-Quintana AA. Cardiovascular Disorders in Hospitalized Patients with Dengue Infection. Enferm Infecc Microbiol Clin. 2021;39(3):115-8. doi: 10.1016/j.eimc.2020.02.032. 10.1016/j.eimc.2020.02.032 32345488

[14] 14 Wei KC, Sy CL, Wang WH, Wu CL, Chang SH, Huang YT. Major Acute Cardiovascular Events after Dengue Infection-A Population-Based Observational Study. PLoS Negl Trop Dis. 2022;16(2):e0010134. doi: 10.1371/journal.pntd.0010134. 10.1371/journal.pntd.0010134 PMC8853534 35130277

[15] 15 Sosothikul D, Seksarn P, Pongsewalak S, Thisyakorn U, Lusher J. Activation of Endothelial Cells, Coagulation and Fibrinolysis in Children with Dengue Virus Infection. Thromb Haemost. 2007;97(4):627-34. 17393026

[16] 16 Pesaro AE, D’Amico E, Aranha LF. Dengue: Cardiac Manifestations and Implications in Antithrombotic Treatment. Arq Bras Cardiol. 2007;89(2):e12-5. doi: 10.1590/s0066-782x2007001400015. 10.1590/s0066-782x2007001400015 17874007

[17] 17 Chao CH, Wu WC, Lai YC, Tsai PJ, Perng GC, Lin YS, et al. Dengue Virus Nonstructural Protein 1 Activates Platelets via Toll-Like Receptor 4, Leading to Thrombocytopenia and Hemorrhage. PLoS Pathog. 2019;15(4):e1007625. doi: 10.1371/journal.ppat.1007625. 10.1371/journal.ppat.1007625 PMC6497319 31009511

[18] 18 Hottz ED, Lopes JF, Freitas C, Valls-de-Souza R, Oliveira MF, Bozza MT, et al. Platelets Mediate Increased Endothelium Permeability in Dengue Through NLRP3-Inflammasome Activation. Blood. 2013;122(20):3405-14. doi: 10.1182/blood-2013-05-504449. 10.1182/blood-2013-05-504449 PMC3829114 24009231

[19] 19 Malavige GN, Ogg GS. Pathogenesis of Vascular Leak in Dengue Virus Infection. Immunology. 2017;151(3):261-9. doi: 10.1111/imm.12748. 10.1111/imm.12748 PMC5461104 28437586

[20] 20 Buijsers B, Garishah FM, Riswari SF, van Ast RM, Pramudo SG, Tunjungputri RN, et al. Increased Plasma Heparanase Activity and Endothelial Glycocalyx Degradation in Dengue Patients Is Associated With Plasma Leakage. Front Immunol. 2021;12:759570. doi: 10.3389/fimmu.2021.759570. 10.3389/fimmu.2021.759570 PMC8722520 34987504

[21] 21 Brasil. Ministério da Saúde. Nota Técnica nº 16/2024-CGLAB/SVSA/MS. Brasília: Ministério da Saúde; 2024.

[22] 22 Sangkaew S, Ming D, Boonyasiri A, Honeyford K, Kalayanarooj S, Yacoub S, et al. Risk Predictors of Progression to Severe Disease During the Febrile Phase of Dengue: A Systematic Review and Meta-Analysis. Lancet Infect Dis. 2021;21(7):1014-26. doi: 10.1016/S1473-3099(20)30601-0. 10.1016/S1473-3099(20)30601-0 PMC8240557 33640077

[23] 23 Oliveira ÉCL, Pontes ERJC, Cunha RV, Fróes ÍB, Nascimento D. Alterações Hematológicas em Pacientes com Dengue. Rev Soc Bras Med Trop. 2009;42(6):682-5. doi: 10.1590/S0037-86822009000600014. 10.1590/s0037-86822009000600014 20209355

[24] 24 Adane T, Getawa S. Coagulation Abnormalities in Dengue Fever Infection: A Systematic Review and Meta-Analysis. PLoS Negl Trop Dis. 2021;15(8):e0009666. doi: 10.1371/journal.pntd.0009666. 10.1371/journal.pntd.0009666 PMC8372965 34407078

[25] 25 Nivetha S, Kumar A, Eshwari K, Shetty A, Adarsha GK, Saravu K. Clinico-Epidemiological Determinants of Severe Dengue in an Endemic District of Coastal Karnataka. Infect Dis. 2025;57(11):1068-77. doi: 10.1080/23744235.2025.2528132. 10.1080/23744235.2025.2528132 40650448

[26] 26 Nie Q, Li M, Liang Q, Ren J, Li T, Peng W, et al. Clinical Features and Laboratory Indicators of Dengue Infection in China: A Retrospective Study of Adult Patients in a Hospital of Traditional Chinese Medicine. Front Med. 2025;12:1624554. doi: 10.3389/fmed.2025.1624554. 10.3389/fmed.2025.1624554 PMC12277289 40692953

[27] 27 Gamakaranage C, Rodrigo C, Samarawickrama S, Wijayaratne D, Jayawardane M, Karunanayake P, et al. Dengue Hemorrhagic Fever and Severe Thrombocytopenia in a Patient on Mandatory Anticoagulation: Balancing two Life Threatening Conditions: A Case Report. BMC Infect Dis. 2012;12:272. doi: 10.1186/1471-2334-12-272. 10.1186/1471-2334-12-272 PMC3514212 23098331

[28] 28 Lim TS, Grignani RT, Tambyah PA, Quek SC. Impact of Dengue-Induced Thrombocytopenia on Mandatory Anticoagulation for Patients with Prosthetic Heart Valves on Warfarin. Singapore Med J. 2015;56(4):235-6. doi: 10.11622/smedj.2015066. 10.11622/smedj.2015066 PMC4415105 25917474

[29] 29 Bannow BTS, Lee A, Khorana AA, Zwicker JI, Noble S, Ay C, et al. Management of Cancer-Associated Thrombosis in Patients with Thrombocytopenia: Guidance from the SSC of the ISTH. J Thromb Haemost. 2018;16(6):1246-9. doi: 10.1111/jth.14015. 10.1111/jth.14015 29737593

[30] 30 Falanga A, Leader A, Ambaglio C, Bagoly Z, Castaman G, Elalamy I, et al. EHA Guidelines on Management of Antithrombotic Treatments in Thrombocytopenic Patients with Cancer. Hemasphere. 2022;6(8):e750. doi: 10.1097/HS9.0000000000000750. 10.1097/HS9.0000000000000750 PMC9281983 35924068

[31] 31 Cesar LAM, Gowdak LHW, Pavanello R, Ferreira JFM, Mioto BM, Poppi NT, et al. Guideline for Chronic Coronary Syndrome - 2025. Arq Bras Cardiol. 2025;122(9):e20250619. doi: 10.36660/abc.20250619. 10.36660/abc.20250619 PMC13400070 41294178

[32] 32 Bhatt S, Gething PW, Brady OJ, Messina JP, Farlow AW, Moyes CL, et al. The Global Distribution and Burden of Dengue. Nature. 2013;496(7446):504-7. doi: 10.1038/nature12060. 10.1038/nature12060 PMC3651993 23563266

[33] 33 Azeredo EL, Monteiro RQ, Pinto LMO. Thrombocytopenia in Dengue: Interrelationship between Virus and the Imbalance between Coagulation and Fibrinolysis and Inflammatory Mediators. Mediators Inflamm. 2015;2015:313842. doi: 10.1155/2015/313842. 10.1155/2015/313842 PMC4427128 25999666

[34] 34 Schaefer TJ, Panda PK, Wolford RW. Dengue Fever. Treasure Island: StatPearls Publishing; 2024.

[35] 35 Byrne RA, Rossello X, Coughlan JJ, Barbato E, Berry C, Chieffo A, et al. 2023 ESC Guidelines for the Management of Acute Coronary Syndromes. Eur Heart J. 2023;44(38):3720-826. doi: 10.1093/eurheartj/ehad191. 10.1093/eurheartj/ehad191 37622654

[36] 36 Vrints C, Andreotti F, Koskinas KC, Rossello X, Adamo M, Ainslie J, et al. 2024 ESC Guidelines for the Management of Chronic Coronary Syndromes. Eur Heart J. 2024;45(36):3415-37. doi: 10.1093/eurheartj/ehae177. 10.1093/eurheartj/ehae177 39210710

[37] 37 Nso N, Nassar M, Zirkiyeva M, Mbome Y, Ngonge AL, Badejoko SO, et al. Factors Impacting Stent Thrombosis in Patients with Percutaneous Coronary Intervention and Coronary Stenting: A Systematic Review and Meta-Analysis. Cureus. 2022;14(4):e23973. doi: 10.7759/cureus.23973. 10.7759/cureus.23973 PMC9089933 35547463

[38] 38 Yang YX, Liu Y, Li XW, Lu PJ, Wang J, Li CP, et al. Clinical Outcomes after Percutaneous Coronary Intervention for Early versus Late and Very Late Stent Thrombosis: A Systematic Review and Meta-Analysis. J Thromb Thrombolysis. 2021;51(3):682-92. doi: 10.1007/s11239-020-02184-7. 10.1007/s11239-020-02184-7 PMC8049931 32691275

[39] 39 Condello F, Spaccarotella C, Sorrentino S, Indolfi C, Stefanini GG, Polimeni A. Stent Thrombosis and Restenosis with Contemporary Drug-Eluting Stents: Predictors and Current Evidence. J Clin Med. 2023;12(3):1238. doi: 10.3390/jcm12031238. 10.3390/jcm12031238 PMC9917386 36769886

[40] 40 Chau KH, Kirtane AJ, Easterwood RM, Redfors B, Zhang Z, Witzenbichler B, et al. Stent Thrombosis Risk Over Time on the Basis of Clinical Presentation and Platelet Reactivity: Analysis from ADAPT-DES. JACC Cardiovasc Interv. 2021;14(4):417-27. doi: 10.1016/j.jcin.2020.12.005. 10.1016/j.jcin.2020.12.005 33516690

[41] 41 Guimarães PO, Franken M, Tavares CAM, Antunes MO, Silveira FS, Andrade PB, et al. Early Withdrawal of Aspirin after PCI in Acute Coronary Syndromes. N Engl J Med. 2025;393(21):2095-106. doi: 10.1056/NEJMoa2507980. 10.1056/NEJMoa2507980 40888723

[42] 42 Rao SV, O’Donoghue ML, Ruel M, Rab T, Tamis-Holland JE, Alexander JH, et al. 2025 ACC/AHA/ACEP/NAEMSP/SCAI Guideline for the Management of Patients with Acute Coronary Syndromes: A Report of the American College of Cardiology/American Heart Association Joint Committee on Clinical Practice Guidelines. J Am Coll Cardiol. 2025;85(22):2135-237. doi: 10.1016/j.jacc.2024.11.009. 10.1016/j.jacc.2024.11.009 40013746

[43] 43 Koo BK, Kang J, Park KW, Rhee TM, Yang HM, Won KB, et al. Aspirin versus Clopidogrel for Chronic Maintenance Monotherapy after Percutaneous Coronary Intervention (HOST-EXAM): An Investigator-Initiated, Prospective, Randomised, Open-Label, Multicentre Trial. Lancet. 2021;397(10293):2487-96. doi: 10.1016/S0140-6736(21)01063-1. 10.1016/S0140-6736(21)01063-1 34010616

[44] 44 Kang J, Park KW, Lee H, Hwang D, Yang HM, Rha SW, et al. Aspirin versus Clopidogrel for Long-Term Maintenance Monotherapy after Percutaneous Coronary Intervention: The HOST-EXAM Extended Study. Circulation. 2023;147(2):108-17. doi: 10.1161/CIRCULATIONAHA.122.062770. 10.1161/CIRCULATIONAHA.122.062770 36342475

[45] 45 Gragnano F, Cao D, Pirondini L, Franzone A, Kim HS, von Scheidt M, et al. P2Y12 Inhibitor or Aspirin Monotherapy for Secondary Prevention of Coronary Events. J Am Coll Cardiol. 2023;82(2):89-105. doi: 10.1016/j.jacc.2023.04.051. 10.1016/j.jacc.2023.04.051 37407118

[46] 46 Valgimigli M, Choi KH, Giacoppo D, Gragnano F, Kimura T, Watanabe H, et al. Clopidogrel versus Aspirin for Secondary Prevention of Coronary Artery Disease: A Systematic Review and Individual Patient Data Meta-Analysis. Lancet. 2025;406(10508):1091-102. doi: 10.1016/S0140-6736(25)01562-4. 10.1016/S0140-6736(25)01562-4 40902613

[47] 47 Liu D, Xu WP, Xu H, Zhao L, Jin DQ. Efficacy and Safety of Clopidogrel versus Aspirin Monotherapy for Secondary Prevention in Patients with Coronary Artery Disease: A Meta-Analysis. Front Cardiovasc Med. 2023;10:1265983. doi: 10.3389/fcvm.2023.1265983. 10.3389/fcvm.2023.1265983 PMC10616297 37915738

[48] 48 CAPRIE Steering Committee. A Randomised, Blinded, Trial of Clopidogrel versus Aspirin in Patients at Risk of Ischaemic Events (CAPRIE). CAPRIE Steering Committee. Lancet. 1996;348(9038):1329-39. doi: 10.1016/s0140-6736(96)09457-3. 10.1016/s0140-6736(96)09457-3 8918275

[49] 49 Patell R, Hsu C, Shi M, Grosso MA, Duggal A, Buller HR, et al. Impact of Mild Thrombocytopenia on Bleeding and Recurrent Thrombosis in Cancer. Haematologica. 2024;109(6):1849-56. doi: 10.3324/haematol.2023.284192. 10.3324/haematol.2023.284192 PMC11141682 37855029

[50] 50 Iyengar V, Patell R, Ren S, Ma S, Pinson A, Barnett A, et al. Influence of Thrombocytopenia on Bleeding and Vascular Events in Atrial Fibrillation. Blood Adv. 2023;7(24):7516-24. doi: 10.1182/bloodadvances.2023011235. 10.1182/bloodadvances.2023011235 PMC10761355 37756539

[51] 51 Thompson A, Fleischmann KE, Smilowitz NR, de Las Fuentes L, Mukherjee D, Aggarwal NR, et al. 2024 AHA/ACC/ACS/ASNC/HRS/SCA/SCCT/SCMR/SVM Guideline for Perioperative Cardiovascular Management for Noncardiac Surgery: A Report of the American College of Cardiology/American Heart Association Joint Committee on Clinical Practice Guidelines. Circulation. 2024;150(19):e351-e442. doi: 10.1161/CIR.0000000000001285. 10.1161/CIR.0000000000001285 39316661

[52] 52 Joglar JA, Chung MK, Armbruster AL, Benjamin EJ, Chyou JY, Cronin EM, et al. 2023 ACC/AHA/ACCP/HRS Guideline for the Diagnosis and Management of Atrial Fibrillation: A Report of the American College of Cardiology/American Heart Association Joint Committee on Clinical Practice Guidelines. J Am Coll Cardiol. 2024;83(1):109-279. doi: 10.1016/j.jacc.2023.08.017. 10.1016/j.jacc.2023.08.017 PMC11104284 38043043

[53] 53 Levy JH, Mamoun N. Direct Oral Anticoagulants and their Antagonists in Perioperative Practice. Curr Opin Anaesthesiol. 2023;36(4):394-8. doi: 10.1097/ACO.0000000000001275. 10.1097/ACO.0000000000001275 37314165

[54] 54 Wickramasinghe W, Alvitigala BY, Perera T, Karunanayake P, Jayasinghe S, Rajapakse S, et al. Rotational Thromboelastometry in Critical Phase of Dengue Infection: Association with Bleeding. Res Pract Thromb Haemost. 2022;6(3):e12704. doi: 10.1002/rth2.12704. 10.1002/rth2.12704 PMC9033942 35475291

[55] 55 Piza FM, Corrêa TD, Marra AR, Guerra JC, Rodrigues RD, Villarinho AA, et al. Thromboelastometry Analysis of Thrombocytopenic Dengue Patients: A Cross-Sectional Study. BMC Infect Dis. 2017;17(1):89. doi: 10.1186/s12879-017-2204-4. 10.1186/s12879-017-2204-4 PMC5248530 28103832

[56] 56 Sureshkumar VK, Vijayan D, Kunhu S, Mohamed Z, Thomas S, Raman M. Thromboelastographic Analysis of Hemostatic Abnormalities in Dengue Patients Admitted in a Multidisciplinary Intensive Care Unit: A Cross-sectional Study. Indian J Crit Care Med. 2018;22(4):238-42. doi: 10.4103/ijccm.IJCCM_486_17. 10.4103/ijccm.IJCCM_486_17 PMC5930527 29743762

[57] 57 Wickramasinghe W, Alvitigala BY, Perera T, Karunanayake P, Jayasinghe S, Rajapakse S, et al. Rotational Thromboelastometry in Critical Phase of Dengue Infection: Association with Bleeding. Res Pract Thromb Haemost. 2022;6(3):e12704. doi: 10.1002/rth2.12704. 10.1002/rth2.12704 PMC9033942 35475291

