# Supplementary material for: Posicionamento sobre o Manejo de Antitrombóticos e Anticoagulantes em Dengue – 2026
Source: Arq Bras Cardiol. 2026 Apr 28;123(4):e20260216. [Article in Portuguese] doi: 10.36660/abc.20260216 (PMC13155784; doi:10.36660/abc.20260216)
Supplement: Translation

# Translation: Position Statement on the Management of Antithrombotic and Anticoagulant Therapy in Dengue – 2026

**DOI:** 10.36660/abc.20260216i

**Authors:** Fabio Grunspun Pitta, Matheus de Oliveira Laterza Ribeiro, Antonio Eduardo Pesaro, Camila Talita Machado Barbosa, Cláudio Humberto Gonçalves Maia, Daniella Motta da Costa Dan, Evelin Meline Lubrigati, Felipe Carvalho Oliveira, João Gabriel Batista Lage, Julia Nobrega de Brito, Leticia Neves Solon Carvalho, Luciana Dornfeld Bichuette, Luka David Lechinewski, Marcelle Gonçalves Henriques Lizandro, Marcelo Bettega, Marco Antonio Freitas de Queiroz Mauricio Filho, Maria Antonieta Albanez A de Medeiros Lopes, Pedro Felipe Gomes Nicz, Pedro Henrique de Moraes Cellia, Sarah Fagundes Grobe, Sayuri Inuzuka, Stefano Garzon, Tannas Jatene, Wilton Francisco Gomes, Carlos Vicente Serrano

**Affiliations:** 1 São Paulo SP Brazil Einstein Hospital Israelita, São Paulo, SP – Brazil; 2 SP Brazil Instituto do Coração InCor, SP – Brazil; 3 Brasília DF Brazil Instituto de Cardiologia e Transplantes do Distrito Federal, Brasília, DF – Brazil; 4 Cariacica ES Brazil Hospital Meridional de Cariacica, Cariacica, ES – Brazil; 5 Curitiba PR Brazil Hospital INC, Curitiba, PR – Brazil; 6 Salvador BA Brazil Hospital Aliança Star – Rede D’Or, Salvador, BA – Brazil; 7 Rio de Janeiro RJ Brazil Hospital Universitário Clementino Fraga Filho da Universidade Federal do Rio de Janeiro, Rio de Janeiro, RJ – Brazil; 8 Recife PE Brazil Hospital dos Servidores do Estado de Pernambuco, Recife, PE – Brazil; 9 Recife PE Brazil Real Hospital Português de Beneficência em Pernambuco, Recife, PE – Brazil; 10 Fortaleza CE Brazil Hospital de Messejana Dr. Carlos Alberto Studart Gomes, Fortaleza, CE – Brazil; 11 Curitiba PR Brazil Pontifícia Universidade Católica do Paraná, Curitiba, PR – Brazil; 12 Curitiba PR Brazil Hospital São Marcelino Champagnat, Curitiba, PR – Brazil; 13 Brasília DF Brazil Hospital DF Star - Rede D’Or São Luiz, Brasília, DF – Brazil; 14 Curitiba PR Brazil Universidade Federal do Paraná, Curitiba, PR – Brazil; 15 Vitória ES Brazil Universidade Federal do Espírito Santo, Vitória, ES – Brazil; 16 Curitiba PR Brazil Hospital Marcelino Champagnat, Curitiba, PR – Brazil; 17 Curitiba PR Brazil Hospital Universitário Cajuru, Curitiba, PR – Brazil; 18 Goiânia GO Brazil Universidade Federal de Goiás, Goiânia, GO – Brazil; 19 Goiânia GO Brazil Einstein Hospital Israelita, Goiânia, GO – Brazil; 20 Curitiba PR Brazil Faculdades Pequeno Príncipe, Curitiba, PR – Brazil

**Table:** 

Position Statement on the Management of Antithrombotic and Anticoagulant Therapy in Dengue – 2026	
The report below lists declarations of interest as reported to the SBC by the experts during the period of the development of these statement, 2025/2026.	
Expert	Type of relationship with industry
Antônio Eduardo Pesaro	Nothing to be declared
Camila Talita Machado Barbosa	Nothing to be declared
Carlos Vicente Serrano Jr.	Nothing to be declared
Cláudio Humberto Gonçalves Maia	Financial declaration Other relationships Employment relationship with the brazilian or international pharmaceutical, orthosis, prosthesis, equipment and implants industry, as well as any employment relationship with health insurance companies or medical audit companies (including part-time jobs) in the year to which your declaration refers: - Medical auditor for a self-managed health insurance provider (health plan for employees of the Legislative Chamber of the Federal District) – Public service.
Daniella Motta da Costa Dan	Financial declaration Other relationships Funding of continuing medical education activities, including travel, accommodation and registration in conferences and courses, from the brazilian or international pharmaceutical, orthosis, prosthesis, equipment and implants industry: - Viatris: Inspra.
Evelin Meline Lubrigati	Nothing to be declared
Fabio Grunspun Pitta	Nothing to be declared
Felipe Carvalho de Oliveira	Nothing to be declared
João Gabriel Batista Lage	Nothing to be declared
Julia Nobrega de Brito	Financial declaration A - Economically relevant payments of any kind made to (i) you, (ii) your spouse/partner or any other person living with you, (iii) any legal person in which any of these is either a direct or indirect controlling owner, business partner, shareholder or participant; any payments received for lectures, lessons, training instruction, compensation, fees paid for participation in advisory boards, investigative boards or other committees, etc. from the brazilian or international pharmaceutical, orthosis, prosthesis, equipment and implants industry: - Bayer: Nubeqa; Daiichi Sankyo: Lixiana. Other relationships Funding of continuing medical education activities, including travel, accommodation and registration in conferences and courses, from the brazilian or international pharmaceutical, orthosis, prosthesis, equipment and implants industry: - Boehringer Ingelheim: Jardiance Duo; Johnson & Johnson: Imbruvica. Performance, in the previous year, as a medical auditor for health insurance companies or the like: - Medical record auditing: Lean Saúde.
Leticia Neves Solon Carvalho	Financial declaration A - Economically relevant payments of any kind made to (i) you, (ii) your spouse/partner or any other person living with you, (iii) any legal person in which any of these is either a direct or indirect controlling owner, business partner, shareholder or participant; any payments received for lectures, lessons, training instruction, compensation, fees paid for participation in advisory boards, investigative boards or other committees, etc. from the brazilian or international pharmaceutical, orthosis, prosthesis, equipment and implants industry: - Novartis: Inclisiran / Dyslipidemia. Other relationships Funding of continuing medical education activities, including travel, accommodation and registration in conferences and courses, from the brazilian or international pharmaceutical, orthosis, prosthesis, equipment and implants industry: - Novartis: Inclisiran / Dyslipidemia; Novo Nordisk: Semaglutide / Obesity.
Luciana Dornfeld Bichuette	Nothing to be declared
Luka David Lechinewski	Financial declaration A - Economically relevant payments of any kind made to (i) you, (ii) your spouse/partner or any other person living with you, (iii) any legal person in which any of these is either a direct or indirect controlling owner, business partner, shareholder or participant; any payments received for lectures, lessons, training instruction, compensation, fees paid for participation in advisory boards, investigative boards or other committees, etc. from the brazilian or international pharmaceutical, orthosis, prosthesis, equipment and implants industry: - Servier: Acertalix/Acertanlo/Acertil/Triplixam; Bayer: Firialta. Other relationships Funding of continuing medical education activities, including travel, accommodation and registration in conferences and courses, from the brazilian or international pharmaceutical, orthosis, prosthesis, equipment and implants industry: - Chiesi: Trimbow; Servier: Acertalix/Triplixam.
Marcelle Gonçalves Henriques Lizandro	Nothing to be declared
Marcelo Bettega	Nothing to be declared
Marco Antonio Freitas de Queiroz Mauricio Filho	Nothing to be declared
Maria Antonieta Albanez Albuquerque de Medeiros Lopes	Financial declaration A - Economically relevant payments of any kind made to (i) you, (ii) your spouse/partner or any other person living with you, (iii) any legal person in which any of these is either a direct or indirect controlling owner, business partner, shareholder or participant; any payments received for lectures, lessons, training instruction, compensation, fees paid for participation in advisory boards, investigative boards or other committees, etc. from the brazilian or international pharmaceutical, orthosis, prosthesis, equipment and implants industry: - Medtronic: Speaker; Boston Scientific: Speaker. Other relationships Funding of continuing medical education activities, including travel, accommodation and registration in conferences and courses, from the brazilian or international pharmaceutical, orthosis, prosthesis, equipment and implants industry: - Boston Scientific: Travel support as a speaker.
Matheus de Oliveira Laterza Ribeiro	Nothing to be declared
Pedro Felipe Gomes Nicz	Financial declaration A - Economically relevant payments of any kind made to (i) you, (ii) your spouse/partner or any other person living with you, (iii) any legal person in which any of these is either a direct or indirect controlling owner, business partner, shareholder or participant; any payments received for lectures, lessons, training instruction, compensation, fees paid for participation in advisory boards, investigative boards or other committees, etc. from the brazilian or international pharmaceutical, orthosis, prosthesis, equipment and implants industry: - Boston Scientific Corporation – IVUS/Rotablator; Edwards Lifesciences – TAVI; Medtronic – TAVI; MicroPort Scientific Corporation – TAVI. Other relationships Funding of continuing medical education activities, including travel, accommodation and registration in conferences and courses, from the brazilian or international pharmaceutical, orthosis, prosthesis, equipment and implants industry: - Laboratório: Área de atuação/Medicamento/Atividade.
Pedro Henrique de Moraes Cellia	Financial declaration A - Economically relevant payments of any kind made to (i) you, (ii) your spouse/partner or any other person living with you, (iii) any legal person in which any of these is either a direct or indirect controlling owner, business partner, shareholder or participant; any payments received for lectures, lessons, training instruction, compensation, fees paid for participation in advisory boards, investigative boards or other committees, etc. from the brazilian or international pharmaceutical, orthosis, prosthesis, equipment and implants industry: - Biolab: evolocumab / dyslipidemia; - Novo Nordisk: semaglutide / diabetes Other relationships Funding of continuing medical education activities, including travel, accommodation and registration in conferences and courses, from the brazilian or international pharmaceutical, orthosis, prosthesis, equipment and implants industry: - Novo Nordisk: semaglutida Any other interest — financial or other — that should be declared considering the position taken in SBC that has not been expressly listed above: - Instituto Pulsare: medical services
Sarah Fagundes Grobe	Financial declaration A - Economically relevant payments of any kind made to (i) you, (ii) your spouse/partner or any other person living with you, (iii) any legal person in which any of these is either a direct or indirect controlling owner, business partner, shareholder or participant; any payments received for lectures, lessons, training instruction, compensation, fees paid for participation in advisory boards, investigative boards or other committees, etc. from the brazilian or international pharmaceutical, orthosis, prosthesis, equipment and implants industry: - Biolab: Doble; Novartis: Sybrava; Ache: dyslipidemia; Servier: Vastarel; Mantecorp: Women's health; Daiichi Sankyo: Nustenti. B - Research funding under your direct/personal responsibility (directed to the department or institution) from the brazilian or international pharmaceutical, orthosis, prosthesis, equipment and implants industry: - Servier: Vastarel; Daiichi Sankyo: Nustendi. Other relationships Funding of continuing medical education activities, including travel, accommodation and registration in conferences and courses, from the brazilian or international pharmaceutical, orthosis, prosthesis, equipment and implants industry: - Servier; Novartis.
Sayuri Inuzuka	Financial declaration A - Economically relevant payments of any kind made to (i) you, (ii) your spouse/partner or any other person living with you, (iii) any legal person in which any of these is either a direct or indirect controlling owner, business partner, shareholder or participant; any payments received for lectures, lessons, training instruction, compensation, fees paid for participation in advisory boards, investigative boards or other committees, etc. from the brazilian or international pharmaceutical, orthosis, prosthesis, equipment and implants industry: - Laboratório: Área de atuação/Medicamento/Atividade. Other relationships Funding of continuing medical education activities, including travel, accommodation and registration in conferences and courses, from the brazilian or international pharmaceutical, orthosis, prosthesis, equipment and implants industry: - Servier: Antihypertensives.
Stefano Garzon Dias Lemos	Financial declaration A - Economically relevant payments of any kind made to (i) you, (ii) your spouse/partner or any other person living with you, (iii) any legal person in which any of these is either a direct or indirect controlling owner, business partner, shareholder or participant; any payments received for lectures, lessons, training instruction, compensation, fees paid for participation in advisory boards, investigative boards or other committees, etc. from the brazilian or international pharmaceutical, orthosis, prosthesis, equipment and implants industry: - Boston Scientific: Speaker and proctor; Abbott: Speaker and proctor. B - Research funding under your direct/personal responsibility (directed to the department or institution) from the brazilian or international pharmaceutical, orthosis, prosthesis, equipment and implants industry: Amplifier: Cardiac imaging analysis for left atrial appendage occlusion.
Tannas Jatene	Nothing to be declared
Wilton Francisco Gomes	Nothing to be declared

## Table of contents

**Executive Summary/Key Messages** 7
**1. Position Statement Presentation and Methods** 7
  **1.1. Methods** 7
  **1.2. Development Process** 7
  **1.3. Nature of the Recommendations** 7
**2. Introduction** 9
**3. Cardiovascular Involvement** 9
**4. Complementary Tests and Prognostic Evaluation** 10
**5. Considerations for the Management of Antithrombotic and Anticoagulant Therapy in Patients with Dengue** 11
**6. Management of Antiplatelet Therapy in Patients with Dengue** 12
  **6.1. Specific Recommendations for the Management of Antiplatelet Therapy in Patients with Dengue and Thrombocytopenia** 12
  **6.2. Angioplasty and Stenting < 30 Days** 12
  **6.3. Angioplasty and Stenting ≥ 30 Days** 13
  **6.4. Other Clinical Scenarios on Monotherapy (ASA or P2Y12)** 13
  **6.5. Resumption of Antiplatelet Therapy** 13
**7. Management of Anticoagulant Therapy in Patients with Thrombocytopenia and Dengue** 13
  **7.1. General Principles** 13
  **7.2. Patients with low Thrombotic Risk** 13
  **7.3. Patients with high Thrombotic Risk** 13
  **7.4. Resumption of Anticoagulant Therapy** 14
**8. Management of Acute Bleeding** 14
  **8.1. General Measures** 14
  **8.2. Management in Antiplatelet Users** 14
  **8.3. Management of Bleeding in Warfarin Users** 15
  **8.4. Management of Bleeding in DOAC Users** 15
**9. Resumption of Therapy after Acute Bleeding** 15
**10. Conclusion** 16
**References** 16

## Executive Summary/Key Messages

- Dengue has a high incidence in Brazil, with substantial public health and clinical impact, particularly during epidemic periods;
- Dengue can lead to thrombocytopenia, platelet dysfunction, and endothelial damage, markedly increasing the risk of bleeding, especially between days 3 and 7 of illness;
- All patients with platelet counts < 50,000/mm³ should be admitted to hospital (Central Illustration);
- Patients who have undergone angioplasty < 30 days constitute the highest thrombotic-risk group, and dual antiplatelet therapy should be continued whenever platelet counts are ≥ 30,000/mm³;
- For angioplasty performed ≥ 30 days prior, management is more flexible, and monotherapy with a P2Y12 inhibitor without acetylsalicylic acid (ASA) can be continued when platelet counts are between 30,000 and 100,000/mm³;
- In secondary prevention using monotherapy (ASA or P2Y12), continue therapy if platelet counts are ≥ 30,000/mm³; discontinue when < 30,000/mm³;
- In oral anticoagulation, continue therapy if platelet counts are ≥ 50,000/mm³; discontinue when < 50,000/mm³;
- Patients at high thrombotic risk (mechanical prosthetic valve, atrial fibrillation with CHA_2_DS_2_-VASc ≥5, moderate/severe mitral stenosis, recent thrombus) should be monitored in a high-dependency care setting;
- When platelet counts are between 30,000 and 50,000/mm³ in high-thrombotic-risk patients, continuous intravenous unfractionated heparin is preferred due to its short half-life and rapid reversibility;
- Resumption of antiplatelet and anticoagulant therapy should occur during the recovery phase, after clinical stabilization, absence of bleeding, and platelet counts above the recommended thresholds, with a gradual reintroduction when appropriate (Table 1).

**Table 1:** Operational summary

**1) Antiplatelet agents – Stenting < 30 days**		
Platelet counts		Approach
	≥ 30.000/mm³	Continue ASA + P2Y12 (prefer clopidogrel)
	< 30.000/mm³	Discontinue both; consider transfusion; intensive monitoring
**2) Antiplatelet agents – Stenting ≥ 30 days**		
Platelet counts		Approach
	≥ 100.000/mm³	Continue DAPT
	30.000–99.999/mm³	Continue P2Y12 inhibitor and discontinue ASA
	< 30.000/mm³	Discontinue both; resume gradually after stability
**3) Other clinical scenarios on monotherapy (ASA or P2Y12)**		
Platelet counts		Approach
	≥ 30.000/mm³	Continue monotherapy
	< 30.000/mm³	Discontinue monotherapy until recovery
**4) Anticoagulation – patients with low thrombotic risk**		
Platelet counts		Approach
	≥ 50.000/mm³	Continue anticoagulation
	< 50.000/mm³	Discontinue anticoagulation; serial monitoring
**5) Anticoagulation – patients with high thrombotic risk**		
Platelet counts		Approach
	30.000–50.000/mm³	Prefer continuous intravenous unfractionated heparin; monitor aPTT
	< 30.000/mm³	Discontinue anticoagulation; frequent monitoring; resume when ≥ 50,000/mm³

aPTT: activated partial thromboplastin time; ASA: acetylsalicylic acid; DAPT: dual antiplatelet therapy.

## 1. Position Statement Presentation and Methods

### 1.1. Methods

This document synthesizes the available evidence to provide practical and standardized guidelines for the management of antiplatelet and anticoagulant therapy in patients with dengue, especially in the presence of thrombocytopenia and increased risk of bleeding.

### 1.2. Development Process

1. A narrative literature review, with structured searches of major biomedical databases (PubMed, Embase, and Cochrane), including observational studies, case series and reports, systematic reviews, and official documents from the Brazilian Ministry of Health;
2. Assessment of indirect evidence, derived from international guidelines and recommendations applicable to the management of thrombocytopenia of other etiologies;
3. Structured discussion and expert consensus, in accordance with standard practices employed in position statements of the Brazilian Society of Cardiology.

### 1.3. Nature of the Recommendations

The recommendations presented herein are predominantly based on expert opinion, supported by indirect evidence, given the absence of randomized clinical trials specifically addressing the management of antiplatelet or anticoagulant therapy in patients with dengue. Clinical decision-making should therefore be individualized, weighing the balance between hemorrhagic risk and thrombotic risk.

**Figure:** DAPT: dual antiplatelet therapy; OAC: oral anticoagulation; PLT: platelets; PTCA: percutaneous transluminal coronary angioplasty; SAPT: single antiplatelet therapy; UFH: unfractionated heparin.

## 2. Introduction

Dengue is an arboviral disease caused by the dengue virus (DENV), of the genus *Flavivirus*, comprising 4 serotypes (DENV-1 to DENV-4).^1,2^ Over the past 5 decades, its global incidence has increased markedly, with more than 500 million infections estimated annually, approximately 96 million of which are symptomatic.^3,4^ The World Health Organization (WHO) classifies dengue as one of the most relevant arboviral threats to public health.

In Brazil, dengue is endemic and, in 2024, produced the largest outbreak ever recorded in Latin America, with a substantial rise in cases compared with 2023, according to the Pan American Health Organization.^5^ Dengue has been a notifiable disease in Brazil since 1961, and recurrent epidemics have been documented since 1986. Between 2008 and 2019, more than 6000 deaths were attributed to the disease.^6^ Its recent expansion is associated with the simultaneous circulation of all 4 serotypes, vector resistance to insecticides, rapid urbanization, inadequate sanitation, and climate change.^6^

Clinical presentation varies widely: while most cases are asymptomatic or mild, approximately 5% of hospitalized patients progress to severe forms.^7^ Dengue infection progresses through 3 phases: febrile, critical, and recovery.^8^

The febrile phase lasts 2 to 7 days and is characterized by sudden high fever (39–40 °C), headache, retro-orbital pain, myalgia, arthralgia, gastrointestinal symptoms, and rash in up to 50% of cases.

The critical phase, occurring between days 3 and 7, typically begins around the time of defervescence and is characterized by increased capillary permeability, hemoconcentration, and risk of hemorrhage and shock. During this phase, tachycardia, bradycardia, arrhythmias, atrioventricular conduction disturbances, myocarditis, and, in severe cases, circulatory shock secondary to plasma leakage may occur.^8^ The WHO recommends the term "severe dengue" for these situations, reserving the term "severe dengue with hemorrhagic manifestations" for cases where major bleeding is present.^8^

Warning signs include severe abdominal pain, persistent vomiting, serosal effusions, postural hypotension, tender hepatomegaly, mucosal bleeding, lethargy or restlessness, and increased hematocrit level.

Severe dengue is defined by the presence of shock, respiratory distress due to pulmonary congestion, severe bleeding, or severe organ impairment — hepatic, neurological (such as seizures, encephalitis, or coma), cardiac (myocarditis, heart failure, cardiogenic shock), or renal (acute renal failure) dysfunction.

During the recovery phase, the extravasated fluid is reabsorbed, with progressive clinical improvement, hemodynamic stabilization, and delayed platelet recovery. A late-onset rash may appear, and adults often experience prolonged fatigue.

The following sections provide more pathophysiological details of the distinct forms of cardiac involvement during dengue infection.

## 3. Cardiovascular Involvement

Cardiovascular involvement in DENV infection is broad and extends beyond myocardial impairment, potentially affecting the cardiac conduction system, vascular structures, autonomic nervous system, interstitium, and pericardium.^9^ Myocarditis has been recognized by the WHO as a manifestation of severe dengue since 2009, with an estimated incidence of 12.4%,^10^ while self-limiting cardiovascular manifestations, which improve during the recovery phase, occur in approximately 15%–20% of severe cases (Figure 1).^11^

**Figure 1:** Viral and immune mechanisms underlying cardiovascular involvement in dengue infection. DENV: dengue virus; NS1: nonstructural protein 1. Source: Adapted from Yacoub et al.^12^

Myocardial dysfunction may result from direct viral effects, but also from indirect mechanisms such as myocardial edema due to capillary leakage, circulating inflammatory mediators, coronary hypoperfusion, and disturbances in calcium homeostasis.^11,12^ When present, pericarditis is often concomitant with myocarditis and driven by capillary leakage, typically without large pericardial effusions. In a prospective Cuban cohort of 427 patients, its incidence was reported as 1.6%.^13^

In addition to direct cardiac effects, dengue infection appears to be associated with an increased risk of acute ischemic events. In a study including more than 65,000 patients, the risk of acute myocardial infarction and stroke increased during the first 2 weeks after infection.^14^

Thrombocytopenia and bleeding have a complex and multifactorial pathophysiology. During the febrile phase, activation of endothelial cells and the hemostatic system occurs, with an increase in markers such as von Willebrand factor antigen and plasminogen activator inhibitor-1 (PAI-1), concomitant with progressive platelet decline.^15^ Reduced platelet production by the bone marrow results, in part, from direct action of the virus on hematopoietic progenitor cells.^16^

Nonstructural protein 1 (NS1) activates platelets via Toll-like receptor 4 (TLR4), leading to thrombocytopenia and bleeding,^17^ while NLRP3 inflammasome activation induces interleukin (IL)-1β release, increasing endothelial permeability.^18^ In the critical phase, elevated levels of pro-inflammatory cytokines, such as tumor necrosis factor-α (TNF-α), IL-6, IL-13, and IL-18, correlate with increased capillary permeability and hemorrhagic risk.^16,19^

Both NS1 and the virus also increase heparanase activity, leading to degradation of heparan sulfate, a key component of the endothelial glycocalyx.^12^ Disruption of this barrier compromises cell-cell junction integrity and promotes plasma leakage.^20^ Thrombocytopenia is further exacerbated by IL-6-mediated platelet activation and tissue factor induction, leading to systemic activation of coagulation.^16^

These mechanisms demonstrate that the interplay between platelet dysfunction, increased capillary permeability, and an exaggerated inflammatory response constitutes the core pathophysiology of thrombocytopenia and bleeding in dengue. This condition represents a particular challenge for the management of patients with heart disease who require antiplatelet or anticoagulant therapy, owing to the direct impact of these agents on the risk of bleeding.

## 4. Complementary Tests and Prognostic Evaluation

In the evaluation of patients with suspected dengue, some tests assist not only in the confirmation of the diagnosis but also in the early identification of individuals at increased risk of progression to severe disease.

Laboratory diagnosis may be established using direct methods, such as viral isolation or reverse transcriptase-polymerase chain reaction (RT-PCR), or indirect methods, such as detection of immunoglobulin M (IgM) antibodies by means of enzyme-linked immunosorbent assay (ELISA), NS1 antigen capture assays, hemagglutination inhibition test, or plaque reduction neutralization test.^21^ The choice of method and optimal timing of sample collection should follow local epidemiological surveillance guidelines.

Because the immune response to dengue does not allow reliable identification of acute infection solely through antibody detection, NS1 antigen rapid test is the most appropriate diagnostic tool during the early phase.^21^ This test is a valuable resource for rapid, individualized clinical decision-making.^22^

Among routine laboratory tests, complete blood count abnormalities are particularly informative. Marked thrombocytopenia, lymphopenia, and leukopenia are associated with an increased risk of bleeding and organ impairment and are observed in approximately 70%, 67%, and 68.3% of patients with severe dengue, respectively.^23^

Table 2 summarizes recommended tests for suspected dengue, and Table 3 highlights key laboratory abnormalities associated with severe disease.

**Table 2:** Tests for suspected dengue

Category	Recommended tests	Finalidade clínica-científica
Blood test	Complete blood count	Identificar leucopenia, trombocitopenia e hemoconcentração
Liver function	AST (GOT), ALT (GPT), bilirubin, albumin	Avaliar possível acometimento hepático associado a dengue
Coagulation	PT (INR), aPTT, fibrinogen	Detectar alterações de coagulação e risco hemorrágico
Renal function and electrolytes	Urea, creatinine, sodium, potassium	Avaliar complicações metabólicas e renais
Specific markers	NS1, serum IgM/IgG, RT-PCR	Confirmação diagnóstica do vírus da dengue
Complementary tests	Blood gas analysis, C-reactive protein	Avaliar gravidade, resposta inflamatória e necessidade de suporte adicional

ALT (GPT): alanine aminotransferase (glutamic-pyruvic transaminase); aPTT: activated partial thromboplastin time; AST (GOT): aspartate aminotransferase (glutamic-oxaloacetic transaminase); IgM/IgG: immunoglobulin M/immunoglobulin G; NS1: dengue virus nonstructural protein 1; PT (INR): prothrombin time (international normalized ratio); RT-PCR: reverse transcriptase-polymerase chain reaction.

**Table 3:** Key laboratory abnormalities associated with severe dengue

Test/Category	Laboratory abnormality/finding	Frequency (%)	Clinical significance and prognosis
Complete blood count	Thrombocytopenia (< 100,000/mm³; < 50,000/mm³)	70–81,9;^24^ 42,6^25^	Strong association with progression to severe forms and risk of bleeding
Complete blood count	Leukopenia/Lymphopenia	68,3;^23^ 22,2–67,2^23-25^	Common finding; leukocytosis can occur in adults with severe disease
Complete blood count	Monocytosis/Atypical lymphocytosis	67^23^	Immune response to viral infection; nonspecific finding
Complete blood count	Normal platelet count with hemorrhagic manifestations	-	Suggestive of platelet dysfunction
Hematocrit	Significant increase compared to baseline	-	A classic sign of hemoconcentration; indicative of an increased risk of shock
Hematocrit	Low hematocrit	-	Suggestive of acute bleeding
Liver function	Elevated transaminases (AST/ALT) (> 400 U/L, peak days 4–6)	AST 81,2–84,9%; ALT 52,5–73,3^25-26^	Indicative of liver involvement; association with increased risk of severe dengue
Liver function	Hypoalbuminemia (< 3.5 g/L)	52,9^25^	Indicative of plasma leakage; independent marker of severity
Coagulation	Increased aPTT	42,9^24^	Suggestive of coagulopathy and risk of bleeding
Coagulation	Prolonged PT (elevated INR)	16,5^24^	Correlation with liver dysfunction and risk of bleeding
Coagulation	Reduced fibrinogen/Slightly elevated D-dimer level	-	Indicative of fibrinolysis and severity
Coagulation	Positive indirect Coombs test	-	Rare; indicative of immune hemolysis
Renal function and electrolytes	Urea, creatinine, sodium, potassium	-	Assessment of renal complications and metabolic disorders
Complementary tests	Blood gas analysis, C-reactive protein	-	Assessment of severity and inflammatory response
Specific markers	NS1, serum IgM/IgG, RT-PCR	-	Diagnostic confirmation of dengue infection

ALT: alanine aminotransferase; aPTT: activated partial thromboplastin time; AST: aspartate aminotransferase; IgM/IgG: immunoglobulin M/immunoglobulin G; INR: international normalized ratio; NS1: dengue virus nonstructural protein 1; PT: prothrombin time; RT-PCR: reverse transcriptase-polymerase chain reaction.

## 5. Considerations for the Management of Antithrombotic and Anticoagulant Therapy in Patients with Dengue

Current recommendations are based on data derived from case reports,^27^ case series,^28^ national guidelines and documents,^7,15^ and expert consensus among the authors of this position statement. Indirect evidence from the management of antithrombotic and anticoagulant therapy in patients with thrombocytopenia of other etiologies, such as cancer, has also been considered.^29-31^

In general, continuation of anticoagulant and antiplatelet therapy is recommended in patients with cancer when platelet counts are above 50,000/mm³,^30^ and the most recent guideline for chronic coronary syndrome supports the possibility of maintaining dual antiplatelet therapy (DAPT) in patients with cancer and platelet counts above 30,000/mm³.^31^

However, since the pathophysiology of dengue involves unique features, such as inflammation, platelet dysfunction, and endothelial dysfunction, which modify the risk of bleeding, specific recommendations for the management of these patients are detailed in the following sections.

## 6. Management of Antiplatelet Therapy in Patients with Dengue

The high incidence of dengue in Brazil, frequently marked by annual epidemics, poses considerable challenges to the management of patients with heart disease receiving antiplatelet or anticoagulant therapy.^32^ The thrombocytopenia characteristic of dengue, combined with endothelial dysfunction and coagulation disorders, substantially increases the risk of bleeding, making the use of hemostasis-interfering drugs a critical component of clinical decision-making.^33^

In this scenario, careful assessment of the risk-benefit ratio of continuing or discontinuing antithrombotic therapy is essential, weighing the risk of bleeding against the potential thrombotic risk resulting from treatment discontinuation. Platelet count, individual thrombotic risk, and the clinical context for antiplatelet therapy constitute the key parameters guiding management decisions.

Dengue has a broad clinical spectrum, ranging from self-limiting febrile illness to severe disease with bleeding, organ impairment, and shock. Thrombocytopenia with platelet dysfunction is a hallmark of the disease, and the highest risk of bleeding occurs during the transition from the end of the febrile phase into the early critical phase, typically between days 3 and 5 and up to 2 days after defervescence.^34^

Patients undergoing angioplasty and stenting require DAPT for a defined period depending on stent type and clinical condition.^35,36^ Discontinuation of antiplatelet agents — often necessary due to dengue-associated thrombocytopenia — may increase the risk of stent thrombosis, a serious event usually presenting as acute myocardial infarction with high mortality.^37,38^ Studies indicate that premature DAPT discontinuation is the strongest predictor of stent thrombosis, with the period of greatest vulnerability occurring within the first 30 days of stent placement.^39,40^

### 6.1. Specific Recommendations for the Management of Antiplatelet Therapy in Patients with Dengue and Thrombocytopenia

We recommend hospitalization for all patients with platelet counts < 50,000/mm³ for clinical and laboratory monitoring.

Management decisions regarding continuation or discontinuation of antiplatelet therapy should follow the guidance below.

### 6.2. Angioplasty and Stenting < 30 Days

Patients in this group are at very high thrombotic risk,^41,42^ requiring caution when considering therapy discontinuation:

- Platelets ≥ 30,000/mm³: continuing ASA + P2Y12 is recommended, preferably clopidogrel;^43-48^
- Platelets < 30,000/mm³: discontinuing both agents is recommended;
- Platelet transfusion may be considered for counts < 30,000/mm³ to allow continuation of monotherapy, particularly in more symptomatic patients.^30^

### 6.3. Angioplasty and Stenting ≥ 30 Days

- Platelets ≥ 100,000/mm³: continuing DAPT is recommended;
- Platelets 30,000–99,999/mm³: continuing P2Y12 and discontinuing ASA is recommended;
- Platelets < 30,000/mm³: discontinuing both agents is recommended.

These recommendations may also be applied to patients with recent acute myocardial infarction under medical treatment or surgical revascularization.

Patients at high ischemic risk (e.g., multiple stents, long lesions, left main disease, true bifurcation lesions, diabetes mellitus, or chronic kidney disease) may require individualized management, with continuation of DAPT considered when platelet counts are ≥ 50,000/mm³.

### 6.4. Other Clinical Scenarios on Monotherapy (ASA or P2Y12)

This includes patients in secondary prevention (those treated medically, post-coronary artery bypass surgery, or who have completed the recommended duration of DAPT):

- Platelets ≥ 30,000/mm³: continuing therapy is recommended;
- Platelets < 30,000/mm³: discontinuing therapy is recommended.

### 6.5. Resumption of Antiplatelet Therapy

- Resumption is recommended only during the recovery phase, in the absence of bleeding and once platelet counts exceed the defined thresholds;
- A gradual reintroduction may be considered, beginning with P2Y12 inhibitor monotherapy, followed by reintroduction of aspirin in patients with a clinical indication.

## 7. Management of Anticoagulant Therapy in Patients with Thrombocytopenia and Dengue

Clinical decision-making should be individualized, with careful consideration of both bleeding and thrombotic risk.

Assessment of the risk of bleeding should take into account:

- Dengue severity;
- History of moderate or severe bleeding within the past 3 months;
- Chronic kidney disease (stage ≥ III);
- Moderate or severe chronic liver disease;
- Active cancer;
- Age > 75 years.

Thrombocytopenia is common in severe dengue. Studies show that platelet counts < 100,000/mm³ are already associated with an increased risk of bleeding.^41,42^

Assessment of thrombotic risk should classify patients as high-risk when they have:^43^

- Mechanical prosthetic valves, particularly in the mitral position;
- Recent thromboembolic events (past 3 months);
- Atrial fibrillation with CHA2DS2-VASc ≥ 5;
- Atrial fibrillation associated with moderate or severe mitral stenosis;
- Recent left ventricular thrombus (past 3 months);
- Antiphospholipid syndrome.

Decisions regarding discontinuing or adjusting anticoagulation should integrate thrombotic risk, degree of thrombocytopenia, type of anticoagulant, and the patient's clinical status.

Important: direct oral anticoagulants (DOACs) are contraindicated in patients with mechanical prosthetic valves or atrial fibrillation associated with moderate or severe mitral stenosis. These patients should receive coumarins (warfarin).^49-53^

### 7.1. General Principles

- Continuing anticoagulation is recommended when platelets ≥ 50,000/mm³ and no bleeding is present;
- Discontinuing anticoagulation is recommended when platelets < 50,000/mm³, with daily monitoring in a hospital setting;
- Using DOACs in patients with mechanical prosthetic valves or moderate/severe mitral stenosis is not recommended;
- Monitoring prothrombin time/international normalized ratio (PT/INR) in patients receiving warfarin is recommended;
- Classifying thrombotic risk as high is recommended in the presence of:
  – Mechanical prosthetic valve (especially mitral);
  – Atrial fibrillation with CHA_2_DS_2_-VASc ≥ 5;
  – Atrial fibrillation with moderate/severe mitral stenosis;
  – Recent left ventricular thrombus;
  – Antiphospholipid syndrome;
  – Thromboembolic event within the past 3 months.

### 7.2. Patients with Low Thrombotic Risk

- Platelets < 50,000/mm³: discontinuing anticoagulation and admitting to hospital is recommended;
- Resuming anticoagulation is recommended when platelets ≥ 50,000/mm³ and no bleeding is present.

### 7.3. Patients with High Thrombotic Risk

- Platelets 30,000–50,000/mm³: replacing oral anticoagulation with continuous intravenous unfractionated heparin (UFH) is recommended:
  – UFH is preferred due to its short half-life and reversibility with protamine;
  – Serial monitoring of activated partial thromboplastin time (aPTT) is recommended.
- Platelets < 30,000/mm³: discontinuing all anticoagulation is recommended;
- For patients receiving warfarin:
  – Initiating UFH is recommended when INR < 2.0.
- For patients receiving DOACs:
  – Initiating UFH 24–48 hours after the last dose is recommended, taking renal clearance into account.

### 7.4. Resumption of Anticoagulant Therapy

- Anticoagulation should be resumed only during the recovery phase, once the following criteria are met:
  – Clinical stabilization;
  – Absence of bleeding;
  – Platelet count ≥ 50,000/mm³.
- In patients receiving warfarin:
  – Consider bridging with low molecular weight heparin (LMWH) or UFH until therapeutic INR is achieved.
- Viscoelastic testing (e.g., ROTEM delta/ROTEM platelet) may be considered in complex cases.

Figures 2 and 3 summarize the management of antiplatelet and anticoagulant therapy across multiple clinical scenarios.

**Figure 2:** Management of antiplatelet therapy in patients with dengue. ASA: acetylsalicylic acid; DAPT: dual antiplatelet therapy; iP2Y12: P2Y12 inhibitor; PLT: platelets.

**Figure 3:** Management of anticoagulant therapy in patients with dengue. ICU: intensive care unit; PLT: platelets; UFH: unfractionated heparin.

## 8. Management of Acute Bleeding

### 8.1. General Measures

- Immediate discontinuation of all antiplatelet agents and anticoagulants is recommended in the presence of moderate or severe bleeding;
- Patient admission to an intensive care unit (ICU) or high-dependency unit is recommended;
- Daily monitoring of platelet count, hemoglobin/hematocrit, PT/INR, aPTT, and renal and hepatic function is recommended;
- Correction of exacerbating factors (acidosis, hypothermia, electrolyte disturbances) is recommended.

### 8.2. Management in Antiplatelet Users

- In patients receiving DAPT, platelet transfusion is recommended:
  – 1 unit/10 kg, or
  – 1–2 concentrates by apheresis;
- Repeating platelet transfusion is recommended if bleeding persists or if severe thrombocytopenia continues;
- Continuous electrocardiographic monitoring may be considered in patients with recent stent placement.

### 8.3. Management of Bleeding in Warfarin Users

- Use of prothrombin complex concentrate (PCC) for immediate reversal is recommended;
- If PCC is unavailable, administration of fresh frozen plasma (FFP) (15 mL/kg) until INR < 1.5 is recommended;
- Administration of 10 mg of vitamin K (oral or intravenous) is recommended;
- Serial monitoring of INR every 6–12 hours is recommended.

### 8.4. Management of Bleeding in DOAC Users

Dabigatran:

- Idarucizumab is recommended as the specific reversal agent;
- If unavailable, activated PCC (aPCC) may be considered.

Factor Xa inhibitors (rivaroxaban, apixaban, edoxaban):

- Andexanet alfa is recommended;
- If unavailable, 4-factor PCC is recommended.

Additional recommendations:

- Close monitoring of urine output, vital signs, and perfusion status is recommended;
- Avoiding nonsteroidal anti-inflammatory drugs is recommended;
- Tranexamic acid may be considered in cases of severe bleeding when no contraindications are present.

Figure 4 below illustrates the management strategies discussed above.

**Figure 4:** Management of bleeding in patients with dengue. ASA: acetylsalicylic acid; DAPT: dual antiplatelet therapy; FFP: fresh frozen plasma; ICU: intensive care unit; iP2Y12: P2Y12 inhibitor; PCC: prothrombin complex concentrate; PLT: platelets.

## 9. Resumption of Therapy after Acute Bleeding

Transitioning warfarin therapy during dengue is particularly challenging, as INR normalization after discontinuation or resumption occurs slowly and often requires bridging with LMWH until therapeutic INR levels are restored. Although platelet count remains the principal criterion guiding anticoagulation management in patients with dengue, emerging tools are being investigated to refine clinical decision-making. Among these, viscoelastic tests, such as ROTEM platelet and ROTEM delta, have gained attention for providing an integrated assessment of platelet function, coagulation factor activity, and fibrinolysis. These tests can identify abnormalities that may not be detected by conventional laboratory evaluations, such as standard coagulation tests.^54,55^

Selective use of these tools may benefit high-thrombotic-risk subgroups who continue to exhibit substantial risk of bleeding, including individuals with refractory bleeding, those requiring intensive care, individuals with recent invasive procedures, or those with severe hepatic dysfunction.^56,57^ Thus, there is a clear need for more objective and validated criteria to guide the safe resumption of anticoagulation, criteria that go beyond the current empirical thresholds based solely on platelet counts (> 50,000 to 75,000/mm³). Integration of clinical data, traditional laboratory findings, and, when available, viscoelastic parameters may eventually enable a more precise, individualized, and safer approach in these complex clinical scenarios.

## 10. Conclusion

The rising incidence of dengue, marked by recurrent epidemics, poses substantial challenges to the clinical management of patients receiving antithrombotic or anticoagulant therapy. Treatment requires a careful approach, weighing thrombotic risk against the potential for hemorrhage. Temporary discontinuation of anticoagulant or antiplatelet therapy may be necessary; however, such decisions should be individualized, considering the severity of infection and the patient's clinical characteristics.

## Data Availability

The data underlying the text of the Position Statement are included in the manuscript.

## List of abbreviations and acronyms

ALT (GPT): Alanine aminotransferase (glutamic-pyruvic transaminase)
aPCC: Activated prothrombin complex concentrate
aPTT: Activated partial thromboplastin time
ASA: Acetylsalicylic acid (aspirin)
AST (GOT): Aspartate aminotransferase (glutamic-oxaloacetic transaminase)
CHA_2_DS_2_-VASc: Congestive Heart Failure, Hypertension, Age ≥75, Diabetes, Stroke, Vascular disease, Age 65–74, Sex category (female) – Score for thromboembolic risk stratification in atrial fibrillation
DAPT: Dual antiplatelet therapy
DENV: Dengue virus
DENV-1 to DENV-4: Dengue virus serotypes 1 to 4
DOAC: Direct oral anticoagulant
ELISA: Enzyme-linked immunosorbent assay
FFP: Fresh frozen plasma
ICU: Intensive care unit
IgM/IgG: immunoglobulin M/immunoglobulin G
IL: Interleukin
IL-6, IL-13, IL-18: Interleukin subtypes (pro-inflammatory cytokines)
INR: International normalized ratio
LMWH: Low molecular weight heparin
NLRP3: NOD-like receptor family, pyrin domain containing 3 (NLRP3 inflammasome)
NS1: Dengue virus nonstructural protein 1
P2Y12: Platelet ADP receptor (antagonist used as an antiplatelet agent)
PAI-1: Plasminogen activator inhibitor-1
PCC: Prothrombin complex concentrate
PLT: Platelets
PT: Prothrombin time
PTCA: Percutaneous transluminal coronary angioplasty
ROTEM: Rotational thromboelastometry
ROTEM delta: Advanced thromboelastometry device
ROTEM platelet: Specific viscoelastic platelet function test
RT-PCR: Reverse transcriptase-polymerase chain reaction
TLR4: Toll-like receptor 4
TNF-α: Tumor necrosis factor alpha
UFH: Unfractionated heparin
WHO: World Health Organization
TTPa: Tempo de tromboplastina parcial ativado
UTI: Unidade de terapia intensiva
